# Influence of extracellular zinc on M1 microglial activation

**DOI:** 10.1038/srep43778

**Published:** 2017-02-27

**Authors:** Youichirou Higashi, Takaaki Aratake, Shogo Shimizu, Takahiro Shimizu, Kumiko Nakamura, Masayuki Tsuda, Toshio Yawata, Tetuya Ueba, Motoaki Saito

**Affiliations:** 1 Department of Pharmacology, Kochi Medical School, Kochi University, Kohasu, Okoh-cho, Nankoku 783-8505, Japan; 2 Institute for Laboratory Animal Research, Kochi Medical School, Kochi University, Kohasu, Okoh-cho, Nankoku 783-8505, Japan; 3 Department of Neurosurgery, Kochi Medical School, Kochi University, Kohasu, Okoh-cho, Nankoku 783-8505, Japan

## Abstract

Extracellular zinc, which is released from hippocampal neurons in response to brain ischaemia, triggers morphological changes in microglia. Under ischaemic conditions, microglia exhibit two opposite activation states (M1 and M2 activation), which may be further regulated by the microenvironment. We examined the role of extracellular zinc on M1 activation of microglia. Pre-treatment of microglia with 30–60 μM ZnCl_2_ resulted in dose-dependent increases in interleukin-1 beta (IL-1β), interleukin-6 (IL-6), and tumour necrosis factor-alpha (TNFα) secretion when M1 activation was induced by lipopolysaccharide administration. In contrast, the cell-permeable zinc chelator TPEN, the radical scavenger Trolox, and the P2X7 receptor antagonist A438079 suppressed the effects of zinc pre-treatment on microglia. Furthermore, endogenous zinc release was induced by cerebral ischaemia–reperfusion, resulting in increased expression of IL-1β, IL-6, TNFα, and the microglial M1 surface marker CD16/32, without hippocampal neuronal cell loss, in addition to impairments in object recognition memory. However, these effects were suppressed by the zinc chelator CaEDTA. These findings suggest that extracellular zinc may prime microglia to enhance production of pro-inflammatory cytokines via P2X7 receptor activation followed by reactive oxygen species generation in response to stimuli that trigger M1 activation, and that these inflammatory processes may result in deficits in object recognition memory.

A large amount of zinc—one of the most essential trace elements in the body—is sequestered into synaptic vesicles of a specific subset of glutamatergic neurons, particularly in the hippocampus of the mammalian brain. In response to physiological neuronal excitation, vesicular zinc is co-released with glutamate into the extracellular space, and research has revealed that zinc homeostasis plays an important role in brain functions such as learning and memory^1^,^2^. On the other hand, in many pathological conditions such as ischaemia and hypoglycaemia, massive amounts of zinc are released, which then accumulate in postsynaptic neurons, resulting in neuronal cell death^3^,^4^,^5^. Recent studies have revealed that extracellular zinc acts to prevent the uptake of glutamate into astrocytes and induce interleukin (IL)-23 expression in a dose-dependent manner, suggesting that presynaptic zinc release mediates the progression of the aforementioned disorders by regulating glial cell functions as well as neuronal cell death^6^,^7^.

Microglia are the resident immune cells of the central nervous system, continuously surveying their local microenvironment by extending/withdrawing their ramifications, even under normal physiological conditions^8^. However, chronic activation of microglia appears to be characteristic of various neuropathological conditions, such as Parkinson’s disease, Alzheimer’s disease, and amyotrophic lateral sclerosis^9^. Many recent studies have demonstrated that activated microglia in ischaemic brains can exert either detrimental or protective effects, suggesting that these cells may acquire opposing phenotypes, which have been termed the M1 and M2 activation states^10^,^11^. Although these states have been implicated in macrophage-driven immunity^11^, the concept of M1/M2 activation remains controversial^12^. M1 activation is generally referred to as the pro-inflammatory and cytotoxic phenotype, characterised by the production of pro-inflammatory cytokines such as IL-1 beta (IL-1β) and IL-6^13^. In contrast, the M2 phenotype is described as an alternative activation state involved in the fine-tuning of inflammation, tissue remodelling, and repair. This diversity in the microglial response is thought to be regulated by factors in the microenvironment^14^,^15^. However, the role of extracellular zinc in the regulation of these microglial phenotypes remains to be elucidated.

Ischaemia results in the immediate release of zinc into the hippocampal extracellular space and is followed by a second release of zinc at the onset of reperfusion^16^. Previously, we demonstrated that extracellular chelatable zinc triggers morphological changes in cultured microglia and the brain following cerebral ischaemia, and that these morphological changes are mediated by zinc uptake, P2X7 receptor activation, and reactive oxygen species (ROS) generation^17^,^18^. On the other hand, drastic post-ischaemic inflammation following the activation of microglia has been associated with secondary expansion of the infarction and deterioration of neurological outcomes. Toll-like receptor 4 (TLR4), which is predominantly expressed in brain microglia, has been observed to participate in such inflammatory responses^19^,^20^. In general, TLR4 plays a key role in the innate mammalian immune response to microbial membrane components such as lipopolysaccharides (LPS), though it is also activated by endogenous ligands, including the products of extracellular matrix breakdown and molecules released from necrotic cells following global ischaemia^21^,^22^. Recent research has demonstrated that the activation of TLR4 by LPS induces the M1 phenotype of microglia, which is characterised by an increase in the expression of pro-inflammatory cytokines and M1 cell-surface markers such as CD16/32^23^. Hu *et al*. revealed that, in the early stages of ischaemic stroke, microglia in peri-infarct regions gradually transform from the M2 phenotype to the M1 phenotype^24^. Furthermore, increasing evidence has indicated that M1 microglia exacerbate neuronal cell death and cognitive impairment^24^,^25^,^26^.

In the present study, we investigated whether and how extracellular zinc affects the secretion of pro-inflammatory cytokines from LPS-stimulated microglia. We hypothesised that endogenous extracellular chelatable zinc would promote inflammatory activity of microglia with the M1 phenotype in the hippocampus following cerebral ischaemia, and that these pro-inflammatory functions would be further mediated by zinc uptake, P2X7 receptor activation, and ROS generation.

## Results

### Effects of zinc on LPS-induced pro-inflammatory cytokine secretion from microglia

**Figure 1:**
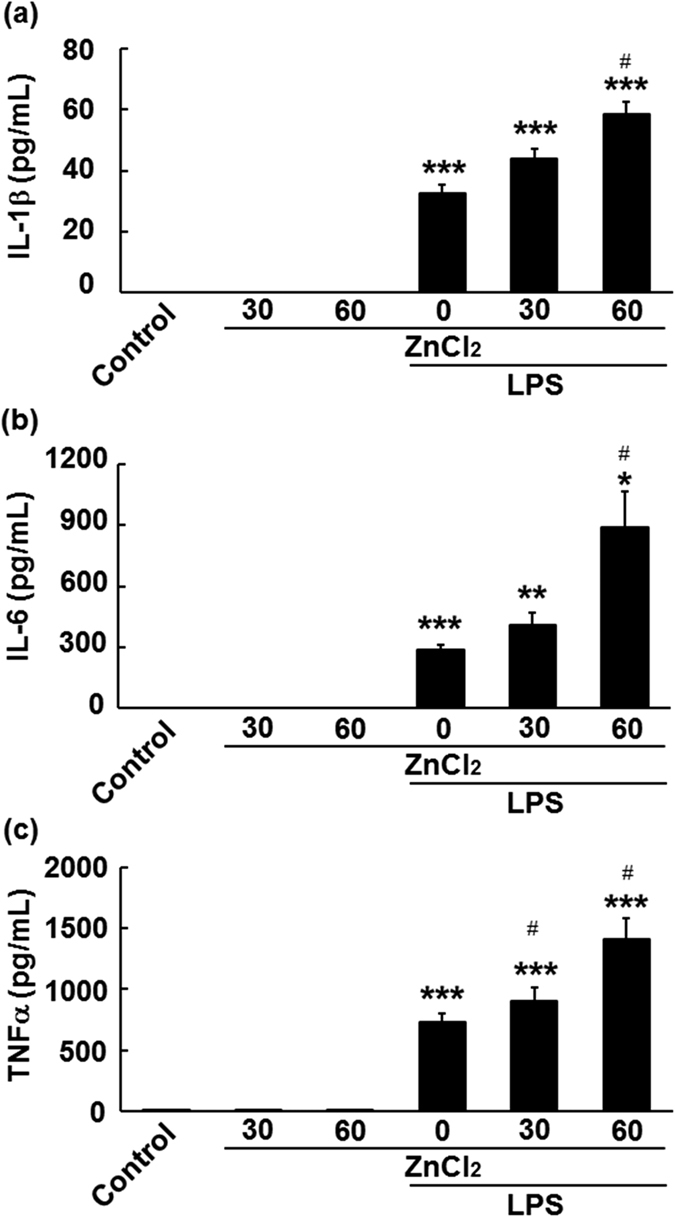
Zinc pre-treatment facilitates the secretion of pro-inflammatory cytokines from lipopolysaccharide (LPS)-stimulated microglia. After microglia had been treated with or without 30 and 60 μM ZnCl_2_ for 2 h, followed by one washout with warmed Eagle’s minimum essential medium, they were stimulated with 1 ng/mL LPS. Levels of interleukin-1 beta (IL-1β) (**a**), interleukin-6 (IL-6) (**b**), and tumour necrosis factor-alpha (TNFα) (**c**) were measured using enzyme-linked immunosorbent assays. The data are presented as the mean ± the standard error of the mean (n = 4). *p < 0.05, **p < 0.001, ***p < 0.005 significantly different from the control group; ^#^*p* < 0.05, significantly different from the group treated with LPS alone.

M1 activation of microglia is characterised by an upregulation of IL-1β, IL-6, and TNFα. To determine whether zinc affects LPS-induced M1 activation, we pre-treated microglia with 30–60 μM ZnCl_2_ prior to LPS stimulation. As depicted in Fig. 1a–c, when microglia were treated with LPS alone, levels of IL-1β (32.74 ± 2.64 pg/mL, p < 0.005), IL-6 (285.42 ± 26.04 pg/mL, p < 0.005), and TNFα (733.63 ± 66.59 pg/mL, p < 0.005) increased significantly, while treatment with zinc alone had no effect on levels of these cytokines. In contrast, pre-treatment with zinc resulted in significant dose-dependent increases in levels of these cytokines in LPS-treated microglia (Fig. 1a–c: in 30 μM ZnCl_2_ + LPS: IL-1β, 43.91 ± 3.00 pg/mL, p = 0.07; IL-6, 411.43 ± 61.37 pg/mL, p = 0.06; TNFα, 907.36 ± 108.50 pg/mL; p < 0.05, in 60 μM ZnCl_2_ + LPS: IL-1β, 58.56 ± 3.97 pg/mL, p < 0.05; IL-6, 885.81 ± 180.56 pg/mL, p < 0.05; TNFα, 1413.48 ± 168.41 pg/mL, p < 0.005). These results indicate that zinc pre-treatment enhanced the LPS-induced secretion of pro-inflammatory cytokines from microglia. Two-way ANOVA revealed significant effects of LPS-stimulation (IL-1β, *F*[1, 18] = 575.008, p < 0.001; IL-6, *F*[1, 18] = 66.142, p < 0.001; TNFα, *F*[1, 18] = 206.456, p < 0.001) and zinc-pre-treatment (IL-1β, *F*[1, 18] = 15.823, p < 0.001; IL-6, *F*[1, 18] = 8.315, p < 0.005; TNFα, *F*[1, 18] = 8.331, p < 0.005). There was also a significant interaction between the LPS-stimulation and zinc-pre-treatment effects (IL-1β, *F*[1, 18] = 15.823, p < 0.001; IL-6, *F*[1, 18] = 8.315, p < 0.005; TNFα, *F*[1, 18] = 8.464, p < 0.005). Therefore, ZnCl_2_ was used in the following *in vitro* experiments at a concentration of 60 μM.

### Zinc-enhanced pro-inflammatory cytokine secretion from LPS-treated microglia was attenuated by intracellular zinc chelation, P2X7 receptor antagonists, and ROS scavengers

**Figure 2:**
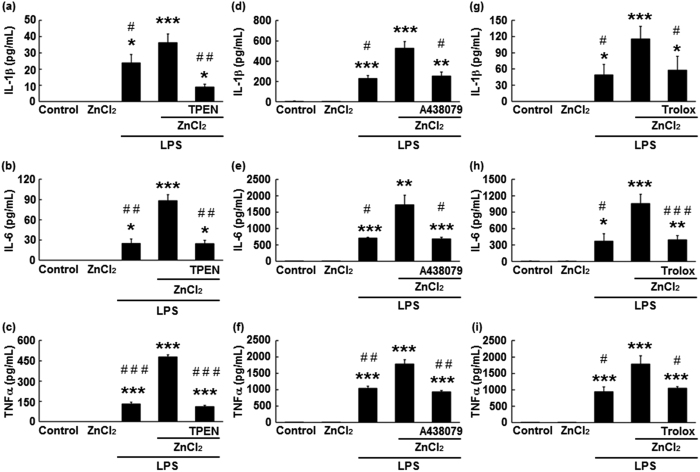
Zinc-enhanced LPS-induced secretion of pro-inflammatory cytokines is mediated by intracellular zinc accumulation, P2X7 receptor activation, and reactive oxygen species generation. (**a**–**c**) After microglia had been treated with or without 1 μM *N,N,N′,N′*-tetrakis(2-pyridylmethyl)ethylenediamine (TPEN) for 30 min, followed by washing with warmed Eagle’s minimum essential medium and 2-h incubation with 60 μM ZnCl_2_, they were treated with 1 ng/mL LPS for 22 h. (**d**–**i**) After microglia had been treated with or without 30 μM A438079 and 500 μM Trolox for 5 min, followed by 2-h incubation with 60 μM ZnCl_2_ and one washout, they were treated with 1 ng/mL LPS for 22 h. The levels of interleukin-1 beta (IL-1β; **a**,**d**, and **g**), interleukin-6 (IL-6; **b**,**e**, and **h**), and tumour necrosis factor-alpha (TNFα; **c**, **f**, and **i**) were measured using enzyme-linked immunosorbent assays. Data are expressed as the mean ± the standard error of the mean (**a**–**c**, n = 3; **d**–**i**, n = 4). *p < 0.05, **p < 0.01, ***p < 0.005, significantly different from the control group; ^#^*p* < 0.05, ^##^*p* < 0.01, ^###^*p* < 0.005, significantly different from the group pre-treated with zinc followed by LPS stimulation. LPS: lipopolysaccharide.

To determine whether zinc uptake is necessary for zinc-enhanced pro-inflammatory cytokine secretion from LPS-treated microglia, microglia were treated with a cell-permeable zinc chelator (TPEN, 1 μM) prior to zinc treatment. Figure 2a–c indicates that pre-treatment of microglia with 1 μM TPEN resulted in significant decreases in levels of IL-1β (p < 0.01), IL-6 (p < 0.01), and TNFα (p < 0.05) 22 h after LPS treatment. Cytokine levels observed after TPEN treatment were almost identical to those observed under LPS treatment alone (Fig. 2b,c: IL-6, p = 0.98, TNFα, p = 0.32). It should be noted that the IL-1β level observed after TPEN treatment was slightly lower than that observed after treatment with LPS alone, although no significant differences were identified between these two groups (Fig. 2a: p = 0.12). In LPS-untreated microglia, TPEN had no effect on pro-inflammatory cytokine secretion (Supplementary Fig. S1). Furthermore, we examined whether TPEN influences cytokine secretion from LPS-stimulated microglia without zinc using the same treatment schedule. Pre-treatment of microglia with TPEN resulted in almost complete suppression of IL-6 secretion 22 h after LPS treatment (Supplementary Fig. S2). Zinc is an essential for ensuring the structural stability or activation of various molecules, such as TNFα-associated factor 6, a key adaptor molecule in the LPS-induced inflammatory response^27^. Research has demonstrated that, in murine macrophages, endogenous intracellular free zinc is involved in TLR signalling via differential regulation of MyD88 and TRIF signalling^28^. Therefore, complete suppression of IL-6 secretion from TPEN-pre-treated microglia without exogenous zinc may have resulted from chelation of intracellular endogenous zinc by TPEN.

Researchers have suggested that the extracellular chelatable zinc uptake process is the first step in producing morphological changes in microglia, via the activation of P2X7 receptors and subsequent ROS generation^17^. In the present study, we also investigated whether P2X7 receptors and ROS generation are involved in the zinc-enhanced secretion of pro-inflammatory cytokines. As indicated in Fig. 2d–i, when microglia were pre-treated with zinc in the presence of a P2X7 receptor antagonist (A438079, 30 μM) and an ROS scavenger (Trolox, 500 μM), the zinc-enhanced secretion of IL-1β, IL-6, and TNFα was significantly attenuated (A438079: IL-1β, p < 0.05; IL-6, p < 0.05; TNFα, p < 0.01; Trolox: IL-1β, p < 0.05; IL-6, p < 0.005; TNFα, p < 0.05). Furthermore, these decreased levels were almost identical to those observed under LPS treatment alone (Fig. 2d–i: A438079: IL-1β, p = 0.71; IL-6, p = 0.59; TNFα, p = 0.12; Trolox: IL-1β, p = 0.69; IL-6, p = 0.80; TNFα, p = 0.35). A previous report by Kappinen *et al*. demonstrated that zinc-induced morphological changes in microglia were inhibited by 100 μM Trolox^18^, although we observed that this lower concentration of Trolox had no effect on zinc-enhanced IL-6 secretion from LPS-treated microglia (Supplementary Fig. S3). Additionally, to confirm that zinc-enhanced pro-inflammatory cytokine secretion was mediated by P2X7 receptor activation and ROS generation, we also performed the same experiments using pyridoxal phosphate-6-azo(benzene-2,4-disulfonic acid)tetrasodium salt hydrate (PPADS), a P2X1-3,5-7 receptor antagonist, and 4-hydroxy-tempo, a low molecular superoxide dismutase mimic. Supplementary Figure S4 shows that, as in the case of A438079, pre-treatment of microglia with PPADS resulted in significant dose-dependent decreases in IL-6 secretion 22 h after LPS treatment. There was no significant difference between secretion of IL-6 from microglia treated with and without 4-hydroxy-tempo, although higher doses of 4-hydroxy-tempo resulted in decreased IL-6 secretion (Supplementary Fig. S4). Furthermore, similar to that observed for TPEN, no significant effects of A438079 or Trolox were observed on the secretion of pro-inflammatory cytokines from LPS-untreated microglia (Supplementary Fig. S1). Pre-treatment of microglia with A438079 or Trolox in the absence of zinc also had no effects on IL-6 secretion from LPS-stimulated microglia (Supplementary Fig. S2).

Because TPEN induces p53-dependent apoptosis in cultured mouse cortical neurons^29^,^30^,^31^, we performed additional experiments using propidium iodide (PI) to determine whether TPEN and other drugs used in the present study affect the cell viability of microglia. As indicated in Supplementary Fig. S5, no significant increases in the number of PI-positive cells pre-treated with zinc in the presence of TPEN or other drugs were observed 22 h following LPS treatment. However, higher doses (120 μM) of zinc resulted in cell death. This observed vulnerability of microglia to ZnCl_2_ is consistent with the findings of a previous report^17^, although Kauppinen *et al*. demonstrated that lower concentrations of ZnCl_2_ (60 μM) also resulted in microglial cell death^18^. These differences may reflect minor variations in cell culture techniques or reagents. In addition, pre-treatment of microglia with TPEN or other drugs had no effect on the viability of LPS-untreated microglia (Supplementary Fig. S5).

We also examined whether zinc-induced P2X7 receptor activation influences microglial proliferation. As shown in Supplementary Figure S6, compared with the control group, we observed no significant differences in the number of PI-negative cells in either the experimental groups or the A438079-treated group, suggesting that zinc-induced activation of P2X7 receptors may be not involved in microglial proliferation.

### Effects of zinc on LPS-induced inducible nitric oxide synthase (iNOS) mRNA expression in microglia

**Figure 3:**
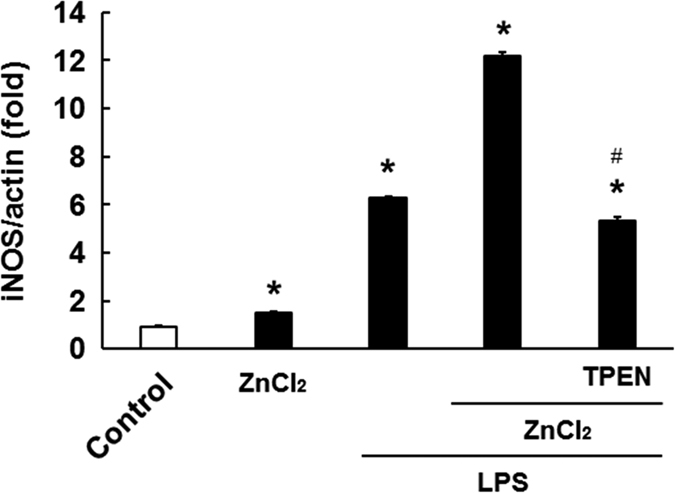
Zinc pre-treatment facilitates the expression of inducible nitric oxide synthase (iNOS) mRNA in LPS-stimulated microglia. After microglia had been treated with or without 60 μM ZnCl_2_ for 2 h, followed by one washout with warmed Eagle’s minimum essential medium, they were stimulated with 1 ng/mL LPS for 6 h. The amount of mRNA for iNOS was normalised to the amount of mRNA for β-actin. The data are presented as the mean ± the standard error of the mean (n = 4). *p < 0.001, significantly different from the control group; ^#^*p* < 0.001, significantly different from the group treated with LPS alone. LPS: lipopolysaccharide.

To confirm that zinc enhances the M1 phenotype of microglia, we evaluated the effects of zinc on the expression of iNOS mRNA (another M1 marker) in LPS-treated microglia. As depicted in Fig. 3, when microglia were treated with LPS alone, the expression of iNOS mRNA increased significantly (6.29 ± 0.06-fold, p < 0.001), while treatment with zinc alone resulted in a small yet significant increase in iNOS mRNA expression (1.50 ± 0.04-fold, p < 0.001). In contrast, pre-treatment with zinc resulted in a significant increase in iNOS mRNA expression in LPS-treated microglia (12.16 ± 0.19-fold, p < 0.001), and this increased expression was almost completely suppressed by TPEN (5.34 ± 0.12-fold, p < 0.001).

### Increased expression of IL-1β, IL-6, and TNFα in the hippocampus following ischaemia–reperfusion was attenuated by intraventricular pre-injection of CaEDTA

**Figure 4:**
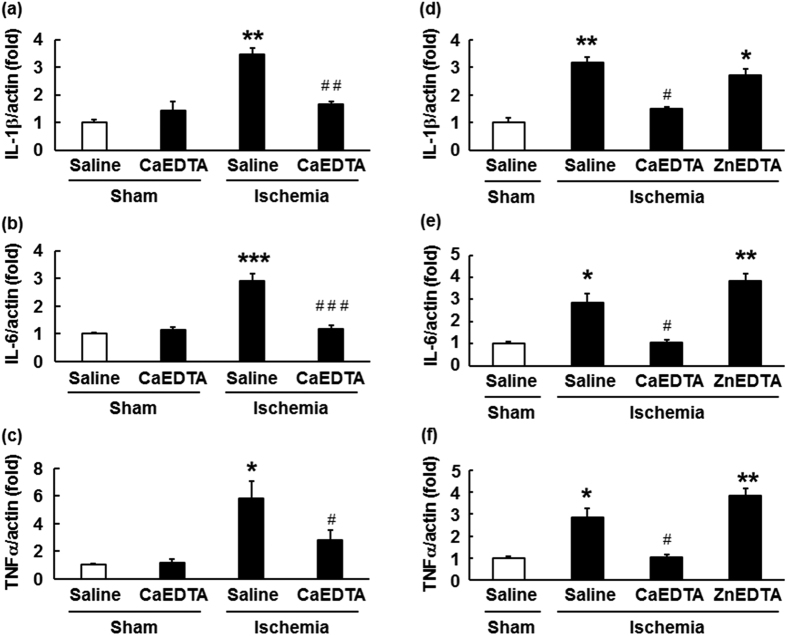
Effects of a zinc chelator on the ischaemia-induced expression of pro-inflammatory cytokines in the hippocampus. (**a–c**) Mice were subjected to transient forebrain ischaemia 5 min after intraventricular injection of a zinc chelator, CaEDTA (300 mM in 2 μL volume). Real-time quantitative polymerase chain reaction was performed using total RNA extracted from the hippocampus of mouse brains 3 days after ischaemia. The amount of mRNA for interleukin-1 beta (IL-1β (**a**), interleukin-6 (IL-6) (**b**), and tumour necrosis factor-alpha (TNFα (**c**) was normalised to the amount of mRNA for β-actin. Data are expressed as the mean ± the standard error of the mean (n = 6). *p < 0.05, **p < 0.01, ***p < 0.001, significantly different from the vehicle-treated sham group; ^#^*p* < 0.05, ^##^*p* < 0.01, ^###^p < 0.005, significantly different from the vehicle-treated ischaemic group. (**d–f**) Mice were subjected to transient forebrain ischaemia 5 min after intraventricular injection of a non-zinc chelator, ZnEDTA (300 mM in 2 μL volume). Real-time quantitative polymerase chain reaction was performed using total RNA extracted from the hippocampus of mouse brains 3 days after ischaemia. The amount of mRNA for IL-1β (**d**), IL-6 (**e**), and was normalised to the amount of (**f**) was normalised to the amount of mRNA for β-actin. Data are expressed as the mean ± the standard error of the mean (n = 4). *p < 0.05, **p < 0.005, significantly different from the vehicle-treated sham group; ^#^*p* < 0.01, significantly different from the vehicle-treated ischaemic group.

Endogenous zinc is released from hippocampal glutamatergic neurons during ischaemia–reperfusion, which triggers morphological changes in microglia^16^,^18^. The effects of extracellular zinc can be blocked with the zinc chelator CaEDTA^3^,^18^. Thus, to evaluate the role endogenous zinc release plays in the expression of hippocampal pro-inflammatory cytokines, we examined the effects of intra-cerebroventricular pre-injection of CaEDTA on the expression of IL-1β, IL-6, and TNFα following brain ischaemia–reperfusion. Three days after ischaemia, the mRNA levels of these cytokines had significantly increased in the hippocampus (IL-1β: 3.46 ± 0.22-fold, p < 0.01; IL-6: 2.9 ± 0.27-fold, p < 0.001; TNFα, 5.81 ± 1.22-fold, p < 0.05), and these increases were significantly suppressed following the administration of CaEDTA (Fig. 4a–c: IL-1b, 1.65 ± 0.11-fold, p < 0.01, IL-6, 1.14 ± 0.14-fold, p < 0.005, TNFα, 2.76 ± 0.76-fold, p < 0.05). The administration of CaEDTA itself resulted in a small increase in cytokine expression, although no significant differences in expression were noted between mice treated with and without CaEDTA. Two-way ANOVA revealed significant effects of ischaemia (IL-1β: *F*[1, 20] = 40.71, p < 0.001; IL-6: *F*[1, 20] = 33.10, p < 0.001; TNFα: *F*[1, 20] = 19.22, p < 0.001) and treatment (IL-1β: *F*[1, 20] = 10.54, p < 0.005; IL-6, *F*[1, 20] = 23.71, p < 0.001). However, no significant effect of CaEDTA treatment on the expression of TNFα mRNA was observed (*F*[1, 20] = 3.904, p = 0.062). We also observed a significant interaction between the effects of ischaemia and treatment (IL-1β: *F*[1, 20] = 28.45, p < 0.001; IL-6: *F*[1, 20] = 31.19, p < 0.001; TNFα: *F*[1, 20] = 4.79, p < 0.05). We observed that the inhibitory effects of CaEDTA were dose-dependent (Supplementary Fig. S7) and sustained for at least 5 days following ischaemia (Supplementary Fig. S8).

We performed additional experiments using ZnEDTA, a non-zinc chelator, to confirm whether chelation of released endogenous zinc suppresses ischaemia-induced pro-inflammatory cytokine expression in the hippocampus. As shown in Fig. 4d–f, intra-cerebroventricular pre-injection of ZnEDTA had no significant effects on the increased expression of these cytokines in the hippocampus of mice 3 days after ischaemia (IL-1β, p = 0.1; IL-6, p = 0.06; TNFα, p = 0.06).

### Effects of CaEDTA pre-injection on M1 microglial polarization in the hippocampus following ischaemia–reperfusion

**Figure 5:**
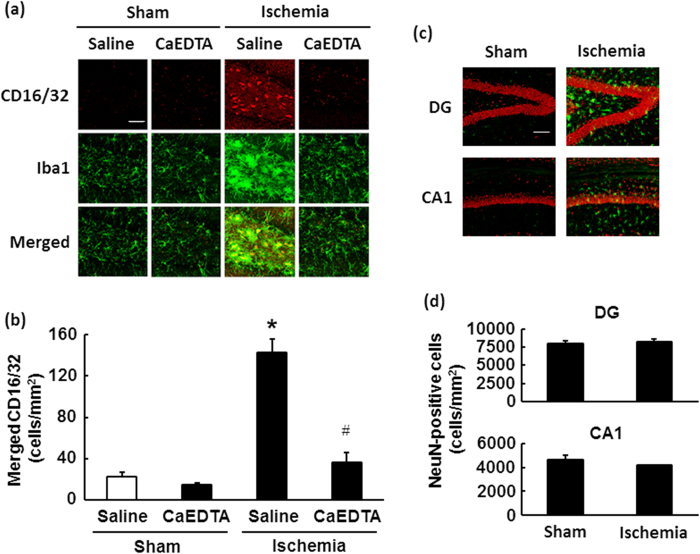
M1 activation of microglia following cerebral ischaemia is blocked by a zinc chelator. Mice were subjected to transient forebrain ischaemia 5 min after intraventricular injection of a zinc chelator, CaEDTA (300 mM in 2 μL volume). (**a**) Representative images of fluorescent double staining of CD16/32 (red) and Iba1 (green) in the hippocampal dentate gyrus region 3 days after ischaemia. Merged images depict CD16/32-positive microglia (yellow). Scale bar = 200 μm. (**b**) Quantification of cells double labelled with CD16/32 and Iba1 in the dentate gyrus. Data are expressed as the mean ± the standard error of the mean (n = 6). *p < 0.05, significantly different from the vehicle-treated sham group; ^#^*p* < 0.05, significantly different from the vehicle-treated ischaemic group. (**c**) Representative merged images of fluorescent double staining of NeuN (red) and Iba1 (green) in the hippocampal dentate gyrus region 3 days after ischaemia. Scale bar = 40 μm. (**d**) Quantification of cells double labelled with NeuN and Iba1 in the dentate gyrus (DG). Data are expressed as the mean ± the standard error of the mean (n = 4).

To evaluate the role endogenous zinc release plays in M1 polarization of microglia in the hippocampus following ischaemia–reperfusion, we analysed the expression of the representative M1 marker CD16/32 via double immunostaining with the microglial marker Iba1. As depicted in Fig. 5a, compared with sham-operated mice, CD16/32 immunoreactivity in Iba1-positive cells was markedly increased in the dentate gyrus (DG) of mice pre-treated with saline 3 days after ischaemia–reperfusion (142.83 ± 12.21 cells/mm^2^, p < 0.001). However, no CD16/32-positive microglia were observed in the ischaemic hippocampal CA1 (Supplementary Figure S9). In contrast, CD16/32 immunoreactivity was very low in the Iba1-positive cells of mice that had received CaEDTA treatment (36.28 ± 9.70 cells/mm^2^, p < 0.001), indicating that extracellular chelatable zinc may be involved in M1 polarization of microglia following transient brain ischaemia (Fig. 5a,b). Two-way ANOVA revealed significant effects of ischaemia (*F*[1, 20] = 75.74, p < 0.001) and treatment (*F*[1, 20] = 50.0, p < 0.001). A significant interaction between the effects of ischaemia and treatment was also observed (*F*[1, 20] = 37.17, p < 0.001). Parallel control experiments were performed to verify the specificity of antibodies against Iba1 and CD16/32 via immunostaining of BV2 cells (a mouse microglial cell line) and T98G cells (a human astrocytic tumour cell line). Iba1- and CD16/32-immunoreactivities were markedly increased in BV2 cells 48 h after 100 ng/mL LPS treatment (Figure S10), consistent with the findings of previous reports^32^,^33^, and the pattern of fluorescence signals was similar that observed in hippocampal microglia 3 days after ischaemia. However, no significant fluorescence was observed in T98 cells regardless of LPS treatment.

We also performed double immunostaining with anti-NeuN and anti-Iba1 antibodies. We observed no significant difference between the number of NeuN-positive cells in either the DG or CA1 of the hippocampus in mice subjected to sham and transient forebrain ischaemia (Fig. 5c,d: sham vs. ischaemia in DG and CA1, respectively: 7978.34 ± 3978.34 cells/mm^2^ vs. 8216.54 ± 348.39 cells/mm^2^, p = 0.693; and 4669.31 ± 336.32 cells/mm^2^ vs. 4158.29 ± 46.56 cells/mm^2^, p = 0.187), indicating that neurons in these regions of C57BL/6 mice were tolerant of transient cerebral ischaemia in the present study. We also observed that many Iba-1-positive microglia exhibited an amoeboid morphology in the DG of the ischaemic hippocampus—characteristic of fully-activated microglia—although very few such cells were observed in the CA1 region (Fig. 4 and Supplementary Figure S9).

### Effects of CaEDTA pre-injection on cognitive dysfunction induced by ischaemia–reperfusion

**Figure 6:**
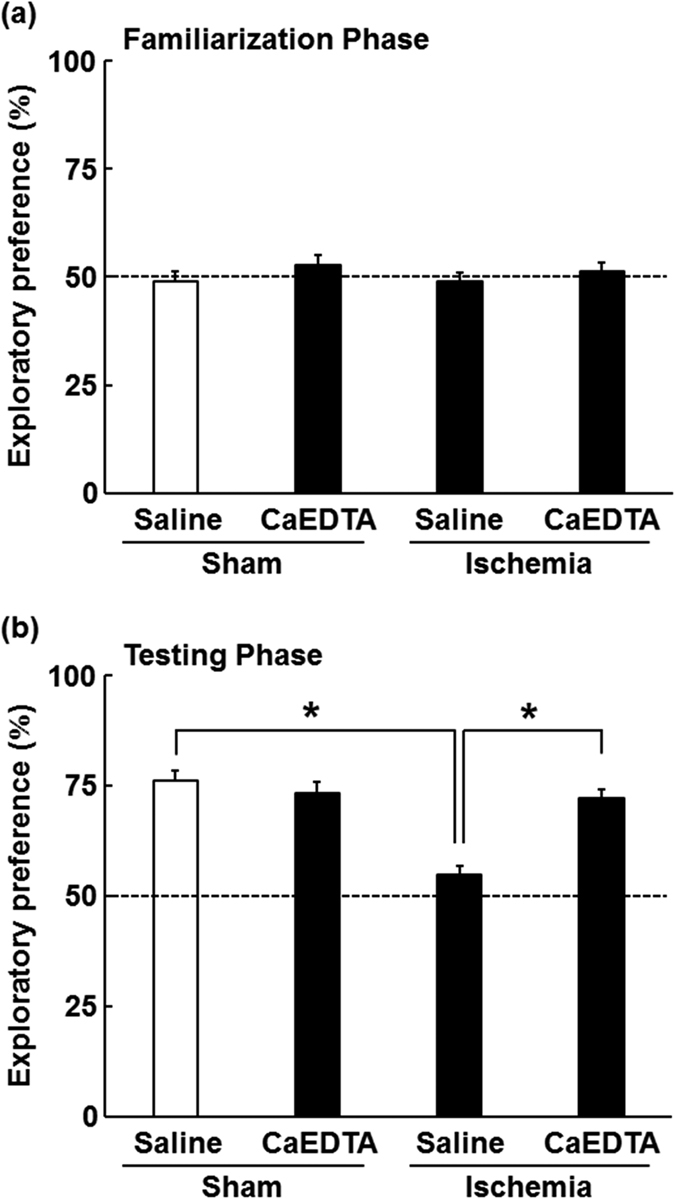
Prevention of ischaemia-induced cognitive deficits via CaEDTA pre-treatment. The object recognition test was performed 10 days after transient forebrain ischaemia. Percentage of preference between two objects in the familiarization phase (**a**) and testing phase (**b**) of the novel object recognition test performed in sham- or ischaemic-operated mice 5 min after vehicle or CaEDTA treatment. Data are expressed as the mean ± the standard error of the mean (n = 6). *p < 0.05, significantly different from the vehicle-treated ischaemic group.

To examine whether intra-cerebroventricular pre-injection with CaEDTA protects against post-ischaemic cognitive decline, we performed a novel object recognition test 10 days after ischaemia–reperfusion. All groups exhibited comparable levels of exploratory behaviour during the familiarisation phase (saline-treated sham-operated mice: 48.96 ± 2.23%, CaEDTA-treated sham-operated mice: 52.69 ± 2.40%, saline-treated ischaemia-operated mice: 48.95 ± 1.91%, CaEDTA-treated ischaemia-operated mice: 51.34 ± 1.96%; Fig. 6a). In the testing phase, both saline- and CaEDTA-treated sham-operated mice exhibited a strong preference for exploration of the novel object when observed for 10 min (saline-treated sham-operated mice: 76.09 ± 3.02%, CaEDTA-treated sham-operated mice: 73.25 ± 1.79%; Fig. 6b). In comparison, saline-treated mice with ischaemia spent almost the same amount of time exploring the familiar object as they did exploring the novel object (54.87 ± 1.84%), indicating the presence of significant cognitive deficits related to object recognition—and therefore hippocampal function—10 days after ischaemia–reperfusion. Strikingly, however, intra-cerebroventricular injection of CaEDTA 5 min prior to ischaemia completely prevented such behaviour (72.10 ± 1.68%): Mice pre-injected with CaEDTA exhibited a preference for the novel object similar to that of the sham-operated mice following ischaemia–reperfusion (Fig. 6b). Two-way ANOVA revealed significant effects of ischaemia (*F*[1, 20] = 26.91, p < 0.001) and treatment (*F*[1, 20] = 11.15, p < 0.001). A significant interaction between the effects of ischaemia and treatment was also observed (*F*[1, 20] = 21.67, p < 0.001).

To examine whether intra-cerebroventricular pre-injection with CaEDTA protects against post-ischaemic deficits in short-term spatial working memory, we also administered a Y-maze test 5 days after ischaemia–reperfusion. As indicated in Supplementary Fig. S11, saline-treated mice with ischaemia exhibited significant decreased in spontaneous alternation behaviour compared with saline-treated sham-operated mice, indicative of impairments in short-term working memory (saline-treated sham-operated mice: 73.19 ± 2.38%, saline-treated ischaemia-operated mice: 62.23 ± 2.03%, p < 0.01). However, intra-cerebroventricular injection of CaEDTA 5 min prior to ischaemia prevented ischaemia-induced reductions in spontaneous alternation behaviour (71.73 ± 4.89%, p < 0.05, Supplementary Fig. S11).

## Discussion

Microglia are morphologically dynamic cells whose alterations are closely associated with their functions^34^. Extracellular chelatable zinc has been demonstrated to transform microglia from their resting form to an activated amoeboid form^20^, though the role of zinc in the regulation of microglial activation states remains poorly understood. The present study demonstrated the following: (1) Extracellular zinc enhanced the LPS-induced secretion of pro-inflammatory cytokines from microglia in a dose-dependent manner; (2) this zinc-induced enhancement was mediated by microglial zinc uptake, P2X7 receptor activation, and ROS generation; (3) microglial uptake of extracellular zinc also enhanced LPS-induced expression of the M1 marker iNOS; (4) CaEDTA, but not ZnEDTA, suppressed ischaemia-induced increases in the expression of pro-inflammatory cytokines and the M1 microglial cell-surface maker CD16/32 in the hippocampus; and (5) CaEDTA protected mice from ischaemia-induced deficits in object recognition memory. These findings suggest that extracellular zinc may be an endogenous factor involved in the promotion of the inflammatory M1 phenotype of microglia in response to M1 stimuli.

Microglia are highly plastic cells that can assume diverse phenotypes and engage in different functions in response to specific signals from the microenvironment^35^. In the maintenance of homeostasis, the initial M1 microglial response is followed by a secondary M2 activation, and this response plays an important role in wound healing and in fine-tuning inflammation^36^,^37^,^38^. In contrast, Kigerl *et al*. reported that the microenvironment associated with spinal cord injuries results in downregulation of the M2 phenotype and upregulation of the M1 phenotype^39^. Furthermore, conditioned medium collected from neurons subjected to oxygen glucose deprivation has been shown to induce M1 polarisation of microglia^24^, indicating that the pathological microenvironment consists of soluble factors released from neurons that may trigger microglial polarisation toward the M1 phenotype. To our knowledge, however, no reports have identified endogenous soluble factors that promote inflammatory activities of the M1 phenotype, including the upregulation of pro-inflammatory cytokine production. In the present study, we observed that extracellular zinc enhanced microglial pro-inflammatory cytokine secretion and iNOS expression, while zinc alone had no effect on the secretion of these cytokines. Several studies have demonstrated increases in extracellular zinc in models of cerebral ischaemia, revealing that treatment with zinc chelators prevents delayed neuronal cell death in the hippocampus following global ischaemia^16^,^40^. These findings suggest that extracellular zinc may prime microglia in the microenvironment to promote the secretion of pro-inflammatory cytokines in response to stimuli associated with M1 activation.

Extracellular chelatable zinc induces morphological changes in microglia following uptake, as well as the subsequent activation of P2X7 receptors and ROS generation via nicotinamide adenine dinucleotide phosphate (NADPH) oxidase activation^17^. Furthermore, the sequential activation of NADPH oxidase, poly(ADP-ribose) polymerase (PARP)-1, and nuclear factor-kappaB (NF-kB) has been implicated in zinc-induced morphological changes to microglia^18^. In the present study, we observed that the zinc-enhanced secretion of pro-inflammatory cytokines from LPS-stimulated microglia was suppressed by the cell permeable zinc chelator TPEN, the P2X7 receptor antagonist A438079, and the ROS scavenger Trolox. In a variety of cell types including microglia, PARP-1 promotes NF-kB transcriptional activity at the promoter sites of target genes by facilitating the binding of NF-kB subunits to DNA^41^,^42^. NF-kB is involved in the expression of several inflammatory mediators including IL-1β, IL-6, and TNFα^43^,^44^,^45^. Therefore, it is possible that the zinc-enhanced secretion of pro-inflammatory cytokines is mediated by NF-kB transcriptional activity via the sequential activation of P2X7 receptors and NADPH oxidase following microglial zinc uptake.

In cultured microglia, TLR4 signalling has been recognized as an essential trigger for M1 polarisation^46^,^47^. Several hours after stroke onset, endogenous TLR4 ligands such as peroxiredoxin and high mobility group box 1 are released from damaged and necrotic cells^48^,^49^. On the other hand, the level of free zinc in the extracellular fluid increases during ischaemia and at the onset of reperfusion^16^. Fluorometric analysis of brain slices has revealed that transient “puffs” or “sparks” of zinc release occur in amounts between 10–30 μM^50^,^51^,^52^. However, direct measurement of free zinc in the extracellular space during cerebral ischaemia has indicated that such elevation occurs only in the nanomolar range (>100 nM, in some cases)^16^, which is much lower than the 60 μM ZnCl_2_ used in cell cultures of the present study. Due to such uncertainties regarding the extracellular concentration of zinc released in the brain during ischaemia, we examined the effect of endogenous zinc release on the M1 phenotype of microglia using the zinc chelator CaEDTA, which has been shown to prevent microglial morphological changes in response to ischaemia-induced releases of zinc^18^. These experiments revealed similar results to the cell culture experiments: Pro-inflammatory cytokine expression increased in the hippocampus after ischaemia, and these increases in expression were attenuated by pre-treatment with CaEDTA but not the non-zinc chelator ZnEDTA. Furthermore, pre-treatment with CaEDTA attenuated ischaemia-induced increases in the expression of the M1 cell surface maker CD16/32. Thus, we considered pre-treatment of microglia with 60 μM ZnCl_2_ 2 h prior to LPS stimulation to be a model of the microenvironmental changes that occur in the hippocampus following ischaemia–reperfusion.

In the present study, we observed CD16/32-immunoreactivity in Iba1-positive cells in the DG, but not in the CA1, of the ischaemic hippocampus. We also observed that Iba1-positive microglia obtained full activated morphology (i.e., amoeboid form) in the DG but not the CA1. Previous studies have revealed that, in response to M1 activation stimuli such as LPS, resting microglia rapidly transform into an amoeboid morphology and exhibit an M1 phenotype^53^. Furthermore, Hu *et al*. reported that the expression of M1-type genes gradually increases over time, beginning on day 3 and continuing for several weeks following ischaemic stroke^24^. These findings may explain the observed increase in CD16/32 immunoreactivity in the limited subfield of the hippocampus 3 days after ischaemia.

Cognitive impairment and neuropsychiatric syndromes are frequent residual consequences of brain ischaemia, and such consequences have a significant effect on an individual’s quality of life and long-term prognosis. Pro-inflammatory cytokines, which are predominantly released from microglia, can affect brain functions such as cognition and emotion^54^,^55^,^56^. The hippocampus is particularly vulnerable to inflammation, and research has indicated that hippocampal inflammation results in learning and memory impairments via reductions in presynaptic glutamate release, neuronal cell death, and suppression of adult hippocampal neurogenesis^57^. On the other hand, cytokines are constitutively expressed at very low concentrations in the healthy brain and are likely involved in normal central nervous system function^58^. Several studies have demonstrated that the effects of ischaemia-induced cortical infarction are exacerbated in mice lacking TNF or p55 receptors for TNFα, and that microglia-driven TNFα activation protects neurons against ischaemic injury^59^,^60^. Furthermore, Yamashita *et al*. reported that the administration of an anti-IL-6 antibody immediately following brain ischaemia results in increased apoptotic cell death and an enlarged infarct size in mice^61^.It is now widely accepted that pro-inflammatory cytokines contribute to repair and recovery after brain ischaemia depending on the timing and degree of their expression. In the present study, the administration of CaEDTA protected mice from ischaemia-induced memory impairments and prevented increases in the expression of pro-inflammatory cytokines and microglial M1 cell-surface markers in the hippocampus following transient forebrain ischaemia, suggesting that excessive inflammation driven from M1 polarised microglia may affect neuronal functions without leading to cell death. However, it is possible that such responses in CaEDTA-pre-treated ischaemic mice are due to the neuroprotective effect of CaEDTA. To evaluate this possibility, neuronal cell viability was assessed by immunostaining using an anti-NeuN antibody. No significant decreases were detected in hippocampal neurons 3 days following transient ischaemia. Research has demonstrated that neuronal cell loss following transient ischaemia produced by occlusion of both common carotid arteries is highly dependent on the mouse strain as well as the duration of the occlusion^62^. For example, previous reports have indicated that the CA1 and DG in C57BL/6 mice are tolerant to such transient ischaemia^62^, in accordance with the results of the present study. Therefore, we suspect that the extracellular chelatable zinc-enhanced production of pro-inflammatory cytokines in M1 polarized microglia may be the primary cause of ischaemia-induced memory impairments, although it is necessary to clarify the precise mechanism by which excessive inflammation influences neuronal functions in future studies.

In summary, extracellular zinc may prime microglia to enhance the secretion of pro-inflammatory cytokines in response to M1 activation stimuli. Such activation may further contribute to object memory impairments in mice subjected to forebrain ischaemia. The results of the present study indicate that the mechanism underlying zinc-enhanced pro-inflammatory activity is mediated by intracellular zinc accumulation and subsequent P2X7 receptor activation, followed by ROS generation. However, a recent clinical trial has revealed that treatment with a zinc chelator is ineffective in patients with acute ischaemic stroke^63^. Accumulating evidence suggests that moderate zinc load is beneficial to neurons even under conditions of ischaemia, whereas excessive amounts of labile zinc are detrimental to neurons^64^,^65^. These findings implicate that interventions targeting microglial zinc-induced signalling pathways may be an effective strategy for preventing brain dysfunction following ischaemia, although it is necessary to clarify the precise mechanism by which released zinc contributes to M1 activation of microglia in the hippocampus following ischaemia in future studies.

## Materials and Methods

Culture plates (6- and 24-well plates) were purchased from Greiner Bio-one (Frickenhausen, Germany). Eagle’s minimum essential medium (EMEM) was purchased from Nissui (Tokyo, Japan). All other chemicals and reagents were obtained from Sigma (St. Louis, MO), except where otherwise noted.

### Animals

Male C57BL/6 mice (8–10 weeks old; Japan SLC, Hamamatsu, Japan) were housed and allowed to habituate for more than 2 weeks prior to the start of experimentation. Food and water were available *ad libitum* throughout the experiments. Animals were maintained in a temperature- (23 ± 1 °C) and humidity-controlled room (55 ± 2%) under a constant day-night rhythm (14/10 h light-dark cycle, lights on at 05:00). All experimental protocols conformed to the guidelines of the National Institutes of Health (Guide for the Care and Use of Laboratory Animals 1996) and were approved by the Committee for the Care and Use of Laboratory Animals at Kochi University.

### Culture of Cells

For microglial cultures, cells were prepared from mixed glial cultures from 1-day-old C57BL/6 mice as previously described^18^, with minor modifications. The cortices from newborn pups were dissociated by mincing and were incubated in papain and DNase for 10 min at 37 °C. After centrifugation for 5 min at 500 × *g*, the cells were re-suspended, triturated with a pipette into EMEM containing 5.6 mM D-glucose, and supplemented with 10% foetal bovine serum (Thermo Trace, Melbourne, Australia), 2 mM glutamine, and 10 mM HEPES. Cells were plated on 6-well plates at a density of 6.4 × 10^5^ cells/well and maintained in a CO_2_ incubator. The medium was changed at 3 days *in vitro* and once per week thereafter. This procedure results in cultures consisting of astrocytes and microglial cells. After 2 weeks *in vitro*, microglia were harvested by mildly shaking the cultures and collecting the floating cells. The cells were re-plated at a density of 2.5 × 10^4^ cells/cm^2^ on 24-well plates to obtain pure microglial cultures. The microglial cultures were used for the experiments 2–3 days after re-plating (*in vitro* days 16–17). Each culture well was visually inspected via phase contrast microscopy prior to use, and wells containing >2% contaminating astrocytes or >30% activated amoeboid microglia were excluded from the experiments, as previously described^18^. At least 3 h prior to the experiments, the culture medium was gently replaced with EMEM.

Mouse BV2 microglial cells (American Type Culture Collection [ATCC], Manassas, VA) and human T98G astrocytoma cells (ATCC) were cultured in Dulbecco’s modified Eagle’s medium containing 5.6 mM D-glucose, supplemented with 10% FBS. For induction of Iba1 and CD16/32 expression, both cell types were treated with 100 ng/mL LPS for 48 h.

### Quantification of cytokines using enzyme-linked immunosorbent assays

Microglia that had been pre-treated with vehicle, 30 μM A438079, 30–100 μM PPADS, 25–50 μM 4-hydroxy-tempo, and 300 μM Trolox for 5 min were subsequently treated with or without the designated concentration of ZnCl_2_ for 2 h. The ZnCl_2_ and respective drugs were washed out once with warmed EMEM, and the cells were then stimulated with 1 ng/mL LPS from *Escherichia coli* 0111 for 22 h. In the case of TPEN, microglia were pre-incubated with 1 μM TPEN for 30 min and subsequently washed once with warmed EMEM prior to ZnCl_2_ treatment. The supernatants of the microglial cultures were then collected. The concentrations of IL-1β, IL-6, and TNFα were measured via enzyme-linked immunosorbent assays according to procedures recommended by the supplier (Biolegend, San Diego, CA). Cell viability of microglia 22 h after LPS treatment was assessed using 1 μg/mL propidium iodide (PI).

### Intraventricular injection of CaEDTA or ZnEDTA and transient ischaemia

C57BL/6 mice were anesthetized with 1–3% isoflurane in a 75:25 mixture of nitrous oxide and oxygen. Mice were given a stereotaxic injection of 2 μL CaEDTA (30–300 mM) or ZnEDTA (300 mM) into the right lateral ventricle (anteroposterior 0.5, mediolateral 1.0, dorsoventral 2.0 mm from bregma and the cortical surface) using a 10 μL Hamilton syringe. The injections were administered over a 5-min period, and the needle was withdrawn after an additional 5 min. Within 5 min after injection of CaEDTA or ZnEDTA, forebrain ischaemia was induced by clamping both common carotid arteries for 20 min^18^. Mice undergoing sham ischaemia received the same surgical incisions and handling without carotid artery occlusion.

Mice were randomly divided into five groups for each treatment: (1) sham plus saline pre-treatment; (2) sham plus CaEDTA pre-treatment; (3) transient ischaemia plus saline pre-treatment; (4) transient ischaemia plus CaEDTA pre-treatment; and (5) transient ischaemia plus ZnEDTA pre-treatment.

### Immunohistochemistry

Mice were sacrificed 3 days after ischaemia. The brains were removed following transcardial perfusion with 0.9% saline and 4% paraformaldehyde. Brains were postfixed in 4% paraformaldehyde overnight and then cryoprotected by immersion in 20% sucrose for 48 h. Coronal sections (30 μm) were prepared and immunostained as previously described^18^. In brief, brain slices were incubated with rabbit anti-mouse Iba1 antibody (dilution 1:500; WAKO, Osaka, Japan), mouse anti-mouse NeuN antibody (dilution 1: 500; Millipore, Schwalbach, Germany), and rat anti-mouse CD16/32 antibody (dilution 1:300; Biolegend) overnight at 4 °C. Antibody binding was visualized with Alexa Fluor 488- and Alexa Fluor 594-labelled secondary antibodies (dilution 1:500; Invitrogen, Carlsbad, CA) using a laser confocal microscope (FV-100D; Olympus, Tokyo, Japan). Negative controls were prepared by omitting the primary antibodies and used to adjust confocal machine settings (i.e., gain and black level). The nuclei were stained with DAPI. Cells labelled with NeuN, or double-labelled with Iba1 and CD16/32, were counted in three representative micrographs of each hippocampal region (bregma −2.20 through −2.66) to quantify the fluorescent signal.

### Quantitative real-time polymerase chain reaction

Total RNA was extracted from the hippocampus using the NucleoSpin RNA Kit (Takara, Otsu, Japan) according to the manufacturer’s instructions, and was then reverse transcribed into cDNA using Oligo-T priming and Moloney murine leukaemia virus reverse transcriptase (Takara). Quantitative polymerase chain reaction was performed using the FastStart Essential DNA Probes Master Mix (Roche, Basel, Switzerland) and run on a Step One Plus Real Time Polymerase Chain Reaction System using the Taqman gene expression assay mix (Applied Biosystems, Carlsbad, CA). Target gene mRNA expression was normalised to β-actin mRNA expression, and the relative amounts of all mRNAs were calculated using the comparative Ct method. The primer sequences and reaction parameters are shown in Table 1.

### Novel object recognition test

The novel object recognition test utilized in the present study is a non-aversive learning paradigm that relies on the spontaneous exploratory behaviour of the studied animals. This method has previously been described in detail^66^. Eight days after ischaemia, mice were habituated to an experimental chamber for 10 min/day for two consecutive days in the absence of an object. The experimental chamber was a cube made of wood (length × width × height: 45 cm × 45 cm × 15 cm) with an open top. Behavioural testing was performed 10 days after ischaemia. On the day of testing, two identical objects were placed in the experimental chamber, and the mouse was allowed to explore the objects for 10 min (“familiarization phase”). After a 1-h delay, the mouse was again placed into the experimental chamber with a single object that was identical to one of the objects used in the familiarisation phase as well as a novel object (“testing phase”). The mice were given 10 min to explore the familiar and novel objects during the testing phase. The familiarisation and testing phases were recorded with a digital video camera. Exploration of the object, defined as the mouse touching the object with its nose and orienting toward the object with the nose within 2.5 cm of the object, was scored cumulatively by two observers who were blinded to the treatment status of each mouse. The results are expressed as a ratio of the time spent exploring either of the two objects during the familiarisation phase or the novel object during the testing phase, divided by the total time spent exploring objects during that phase.

### Spontaneous alternation in a Y-maze test

Short-term working memory was assessed at 5 days following transient ischaemia by recording spontaneous alternation behaviour in a Y-maze. The maze was constructed of grey wood with three identical arms (40 cm × 2 cm × 3 cm) positioned at equal angles. Mice were placed at the end of one arm and allowed to move freely through the maze during a 10-min session. The series of arms entries was recorded with a digital video camera. Alternation was defined if the mouse entered an arm different from the previous two, while an error was defined if the mouse re-entered one of the two previously visited arms. The percentage of relative alternation was calculated from the ratio of the number of alternations divided by the number of total arm entries −2. The value was multiplied by 100.

### Statistical analysis

All data are expressed as the mean ± the standard error of the mean. Comparisons between two or more groups were performed using unpaired *t*-tests or analyses of variance, followed by Fisher’s PLSD test, respectively. Differences with a p-value of 0.05 or less were considered statistically significant. We confirmed the normal distribution of our data using Stat View 5.0 software (Abacus Concepts Inc., Berkeley, CA).

## Additional Information

**How to cite this article:** Higashi, Y. *et al*. Influence of extracellular zinc on M1 microglial activation. *Sci. Rep.* **7**, 43778; doi: 10.1038/srep43778 (2017).

**Publisher's note:** Springer Nature remains neutral with regard to jurisdictional claims in published maps and institutional affiliations.

## Supplementary Material

Supplementary Information

## Figures and Tables

**Table 1:** Primer sets for real-time PCR amplification of proinflammatory cytokines.

Accession number		Primer sequences	Annealing temperature
IL-1β	NM_008361	Forward 5′-CTCTTGTTGATGTGCTGCTG-3′	60 °C
		Reverse 5′-GACCTGTTCTTTGAAGTTGACG-3′	
iNOS	NM_ 010927	Forward 5′-CACTTCTGCTCCAAATCCAA-3′	60 °C
		Reverse 5′-GACTGAGCTGTTAGAGACACTT-3′	
IL-6	NM_031168	Forward 5′-TCCTTAGCCACTCCTTCTGT-3′	60 °C
		Reverse 5′-AGCCAGAGTCCTTCAGAGA-3′	
TNFα	NM_013693	Forward 5′-TCTTTGAGATCCATGCCGTTG-3′	60 °C
		Reverse 5′-AGACCCTCACACTCAGATCA-3′	
β-actin	NM_007393	Forward 5′-GACTCATCGTACTCCTGCTTG-3′	60 °C
		Reverse 5′-GATTACTGCTCTGGCTCCTAG-3′	

IL-1β: interleukin 1 beta; iNOS: inducible nitric oxide synthase; IL-6: interleukin 6; TNFα: tumour necrosis factor alpha; β-actin: beta actin.
