# Supplementary material for: Influence of extracellular zinc on M1 microglial activation
Source: Sci Rep. 2017 Feb 27;7:43778. doi: 10.1038/srep43778 (PMC5327400; doi:10.1038/srep43778)
Supplement: Supplementary Information [file srep43778-s1.pdf]

Supplementary information

Influence of extracellular zinc on M1 microglial activation

Youichirou Higashi, Takaaki Aratake, Shogo Shimizu, Takahiro Shimizu, Kumiko Nakamura, Masayuki Tsuda, Toshio Yawata, Tetuya Ueba, and Motoaki Saito

Contents

Supplementary Figures S1-11

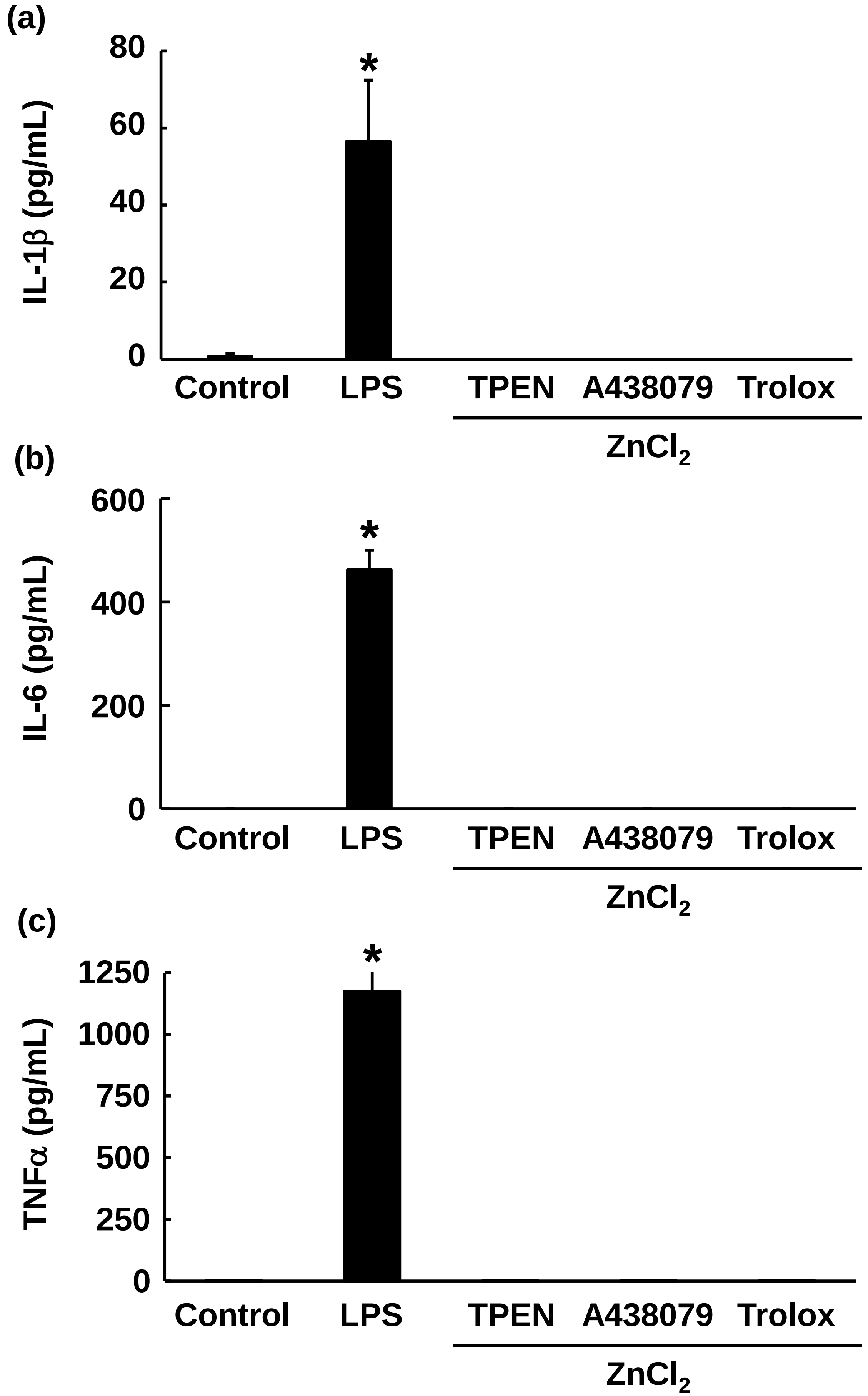

Supplementary Figure S1

Supplementary Figure S1

Secretion of pro-inflammatory cytokines from lipopolysaccharide (LPS)-untreated microglia. After microglia had been treated with or without 1 μM *N*,*N*,*N'*,*N'*-tetrakis(2-pyridylmethyl)ethylenediamine (TPEN) for 30 min, 30 μM A438079 for 5 min, or 500 μM Trolox for 5 min, they were washed with warmed Eagle's minimum essential medium and incubated with 60 μM $ZnCl_2$ for 2 h. They were then treated with or without 1 ng/mL LPS for 22 h. The levels of interleukin-1 beta (IL-1β; **a**), interleukin-6 (IL-6; **b**), and tumour necrosis factor-alpha (TNFα; **c**) were measured using enzyme-linked immunosorbent assays. Data are expressed as the mean ± the standard error of the mean (n = 4). *$p < 0.05$, significantly different from the control group.

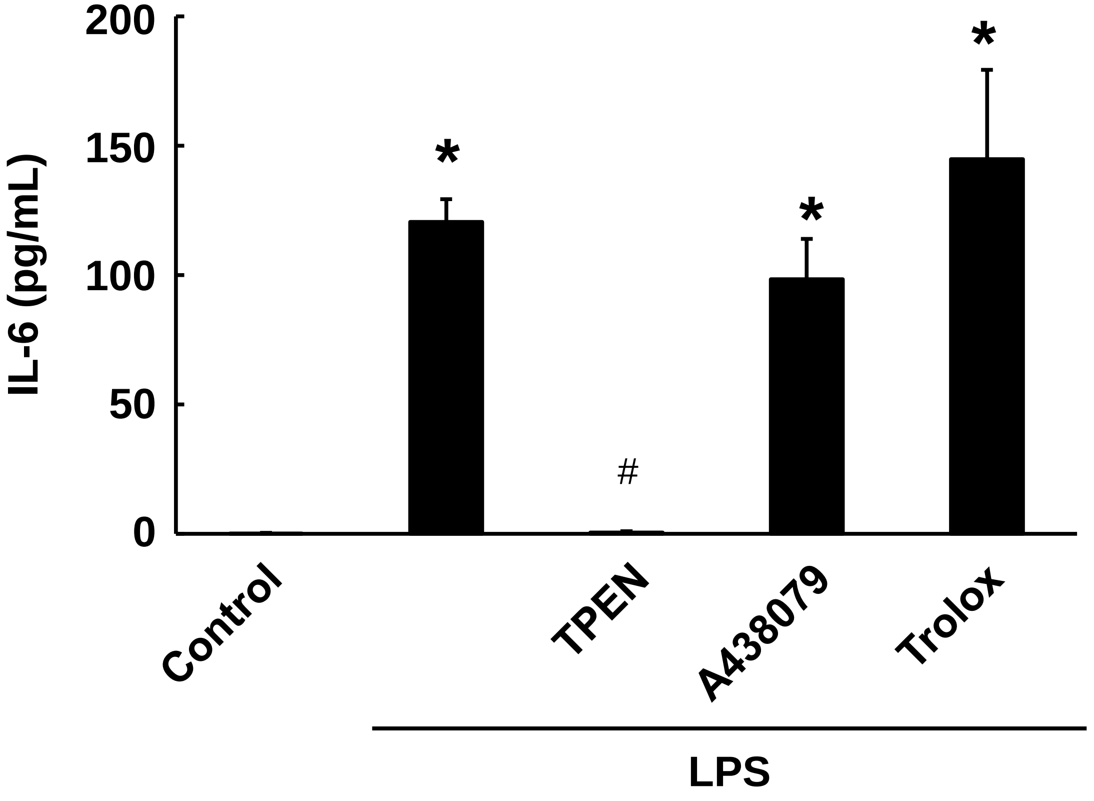

Supplementary Figure S2

Supplementary Figure S2

The effects of TPEN, A438079, and Trolox on LPS-induced IL-6 secretion from microglia. After microglia had been treated with or without 1 μM TPEN for 30 min, 30 μM A438079 for 5 min, or 500 μM Trolox for 5 min, they were washed with warmed Eagle's minimum essential medium and treated with or without 1 ng/mL LPS for 22 h. Levels of IL-6 were measured using enzyme-linked immunosorbent assays. Data are expressed as the mean ± the standard error of the mean (n = 3). $*p < 0.05$, significantly different from the control group. $^{\#}p < 0.05$, significantly different from the group pre-treated with zinc followed by LPS stimulation. TPEN: *N*,*N*,*N'*,*N'*-tetrakis(2-pyridylmethyl)ethylenediamine; LPS: lipopolysaccharide; IL-6; interleukin-6.

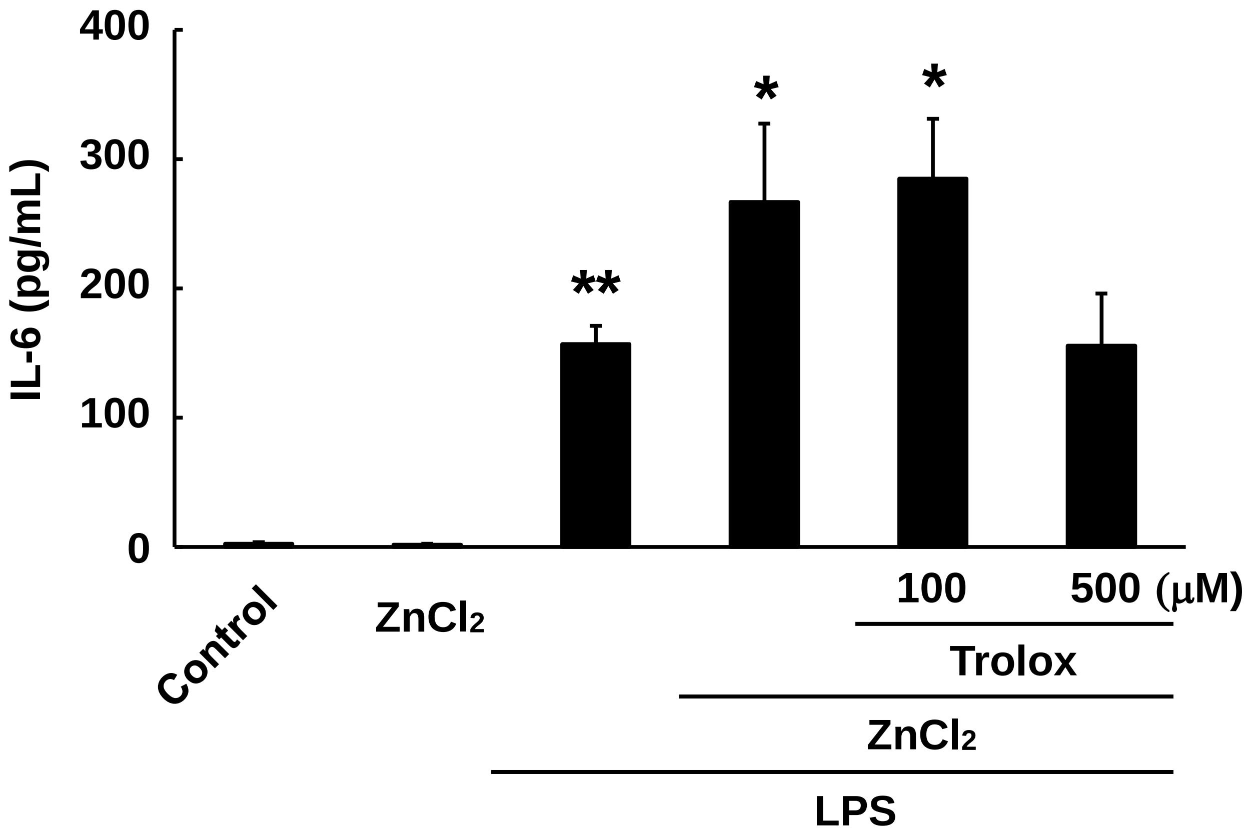

Supplementary Figure S3

Supplementary Figure S3

Zinc-enhanced IL-6 secretion from LPS-treated microglia was not inhibited by lower concentrations of Trolox. After microglia had been treated with or without 100-500 μM Trolox for 5 min, followed by 2-h incubation with 60 μM $ZnCl_2$ and one washout, they were treated with 1 ng/mL LPS for 22 h. Levels of IL-6 were measured using enzyme-linked immunosorbent assays. Data are expressed as the mean ± the standard error of the mean (n = 3). *$p < 0.05$, **$p < 0.01$, ***$p < 0.001$ significantly different from the control group. LPS: lipopolysaccharide; IL-6; interleukin-6.

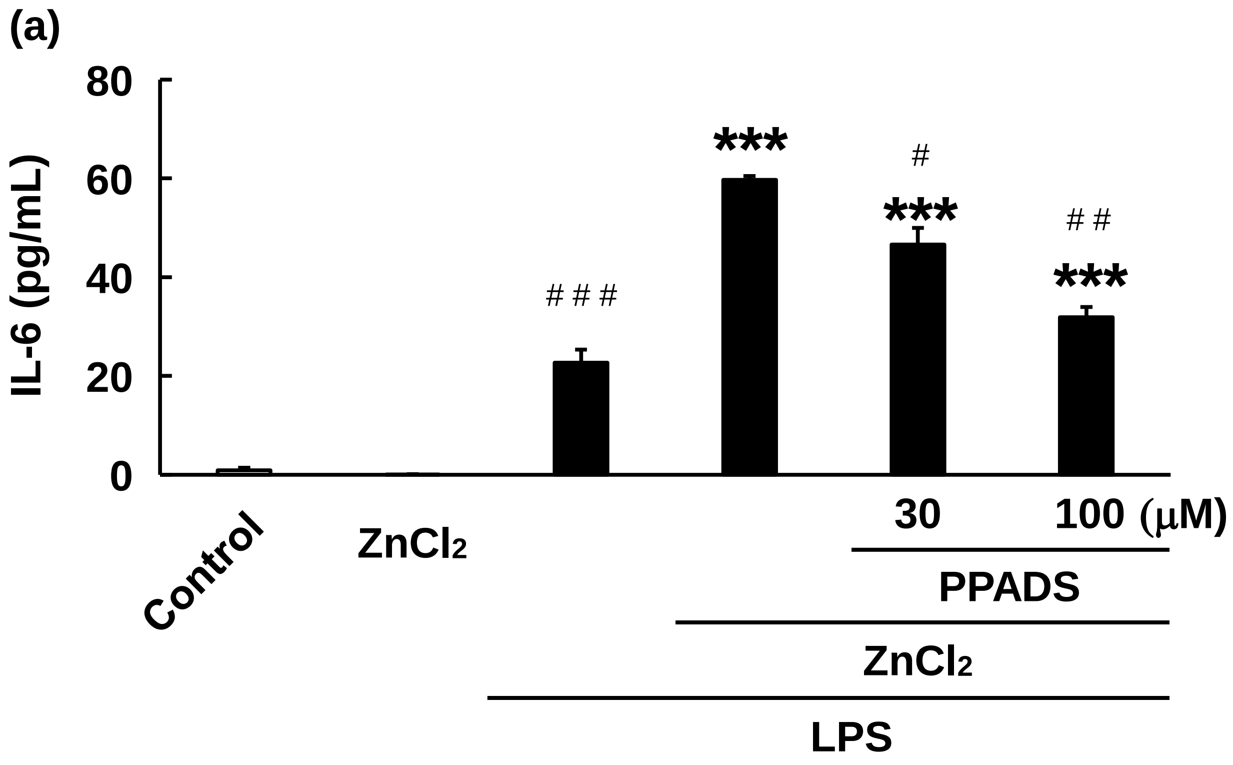

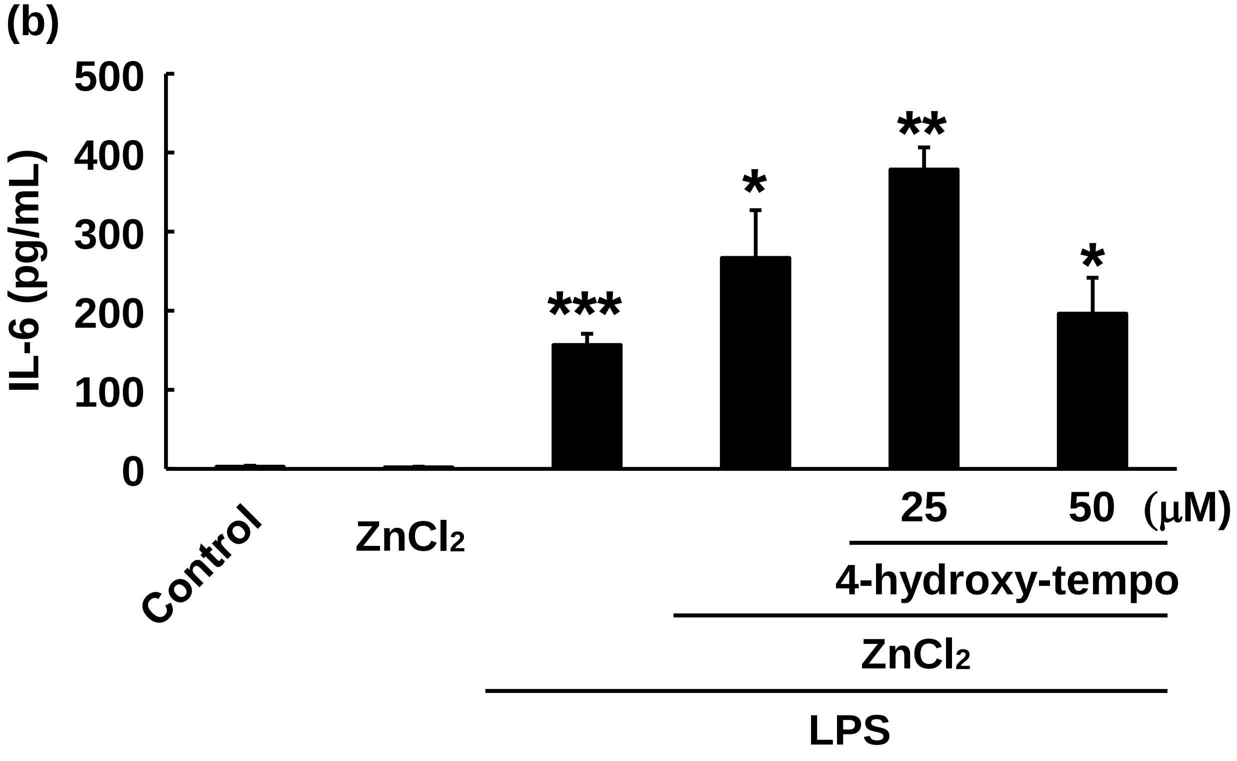

Supplementary Figure S4

Supplementary Figure S4

The effects of pyridoxal phosphate-6-azo(benzene-2,4-disulfonic acid)tetrasodium salt hydrate (PPADS), a P2X1-3, 5-7 receptor antagonist, and 4-hydroxy-tempo , a ROS scavenger, on zinc-enhanced IL-6 secretion from LPS-treated microglia. After microglia had been treated with or without 30-100 μM pyridoxal phosphate-6-azo(benzene-2,4-disulfonic acid)tetrasodium salt hydrate (PPADS) (**a**) or 25-50 μM 4-hydroxy-tempo (**b**) for 5 min, followed by 2-h incubation with 60 μM $ZnCl_2$ and one washout, they were treated with 1 ng/mL LPS for 22 h. Levels of IL-6 were measured using enzyme-linked immunosorbent assays. Data are expressed as the mean ± the standard error of the mean (n = 3). $^{*}p < 0.05$, $^{**}p < 0.01$, $^{***}p < 0.001$ significantly different from the control group; $^{\#}p < 0.05$, $^{\#\#}p < 0.01$, significantly different from the group pre-treated with zinc followed by LPS stimulation.

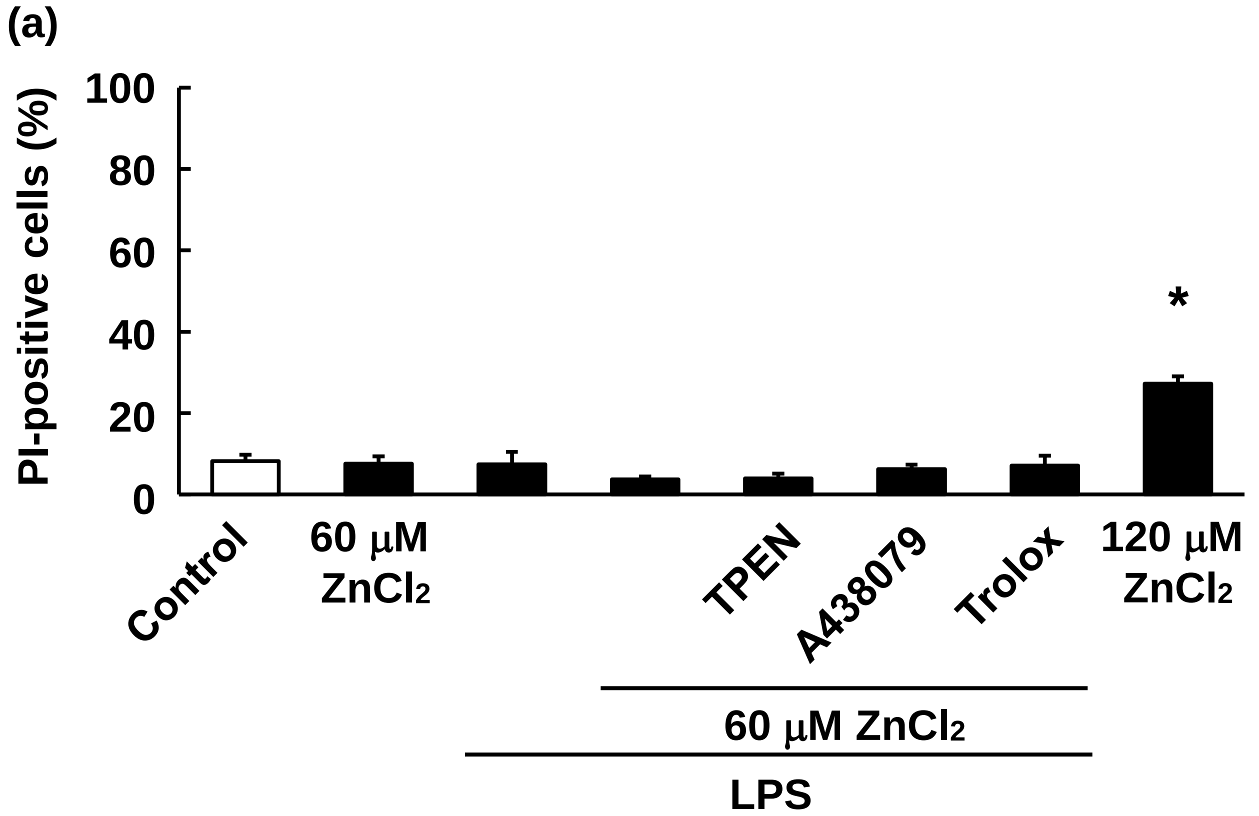

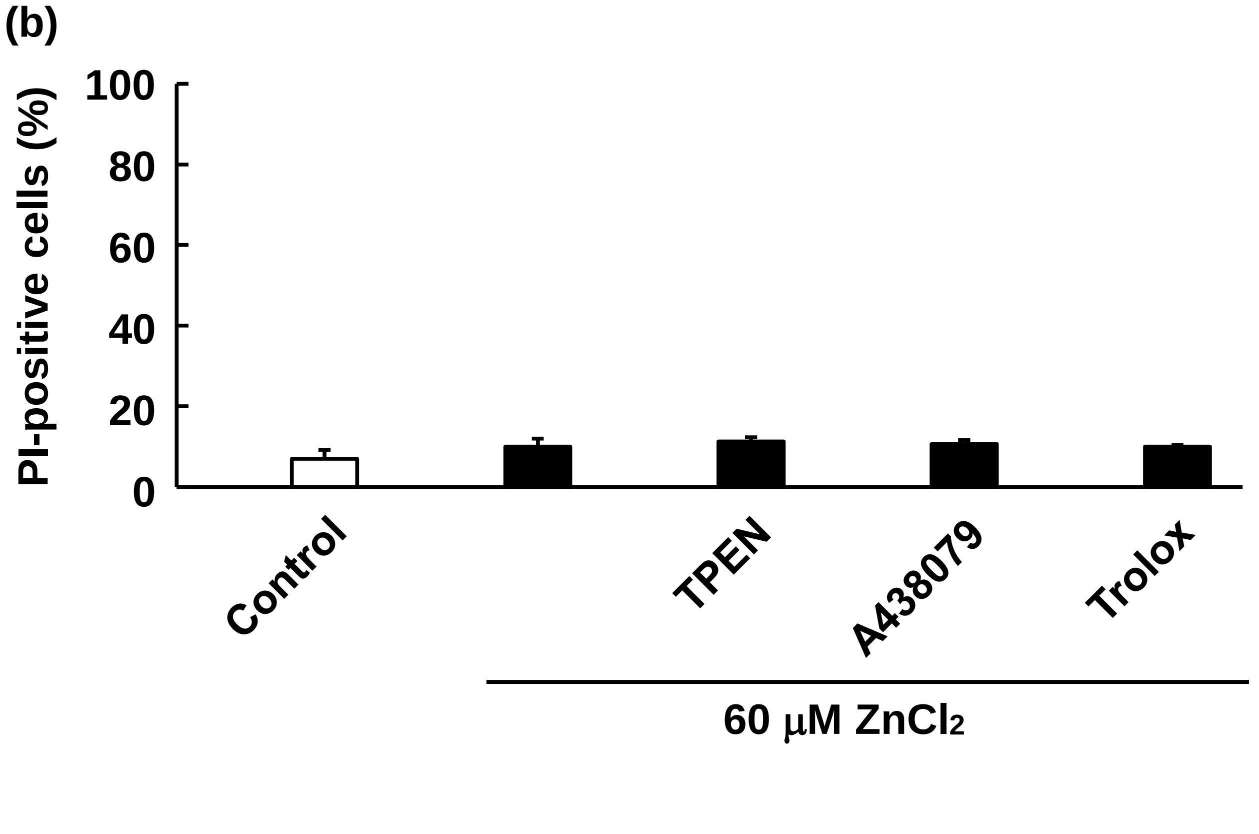

Supplementary Figure S5

Supplementary Figure S5

Microglial cell viability. After microglia had been treated with or without 1 μM TPEN for 30 min, 30 μM A438079 for 5 min, or 500 μM Trolox for 5 min, they were washed with warmed Eagle's minimum essential medium and incubated with 60-120 μM $ZnCl_2$ for 2 h. They were then treated with (**a**) or without (**b**) 1 ng/mL LPS for 22 h. Cell viability as evaluated by cell counting through propidium iodide (PI) staining. Data are expressed as the mean ± the standard error of the mean (n = 3). *$p < 0.05$, significantly different from the control group. TPEN: *N*,*N*,*N'*,*N'*-tetrakis(2-pyridylmethyl)ethylenediamine; LPS: lipopolysaccharide.

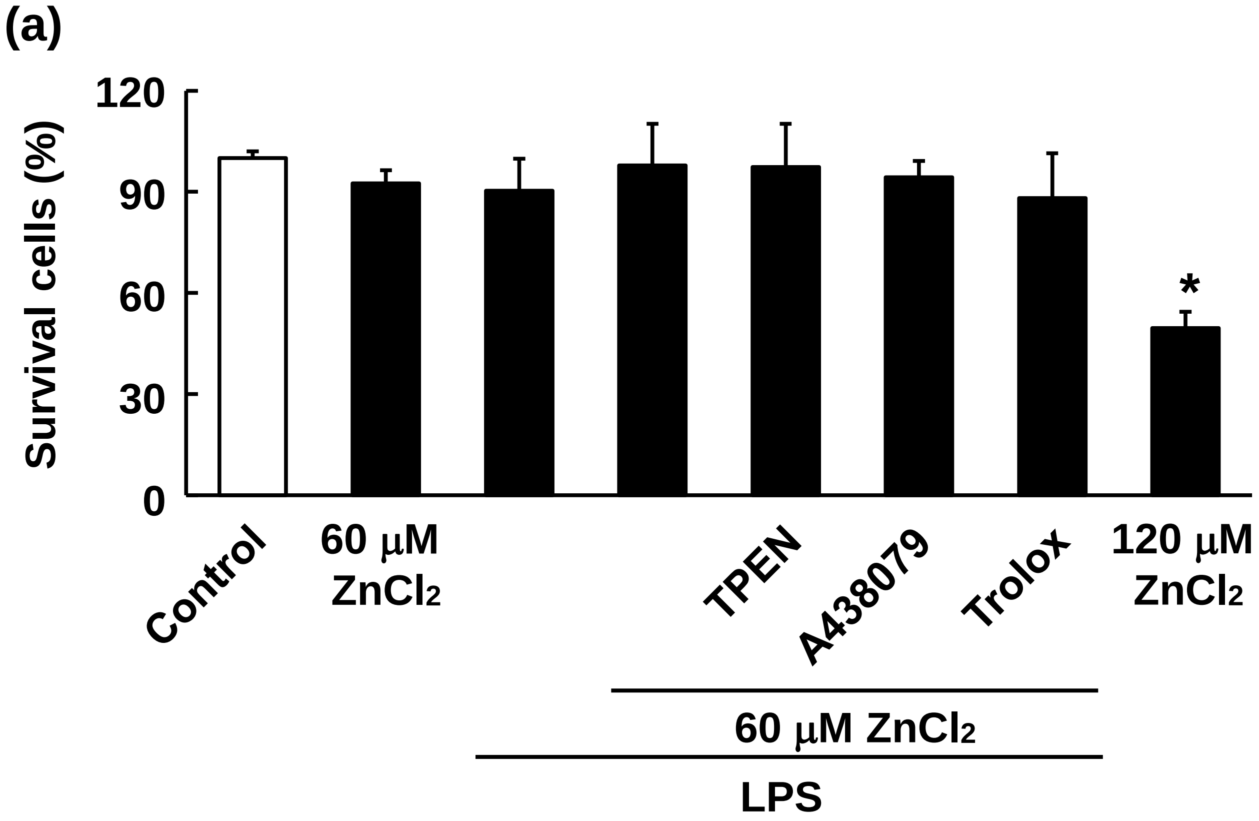

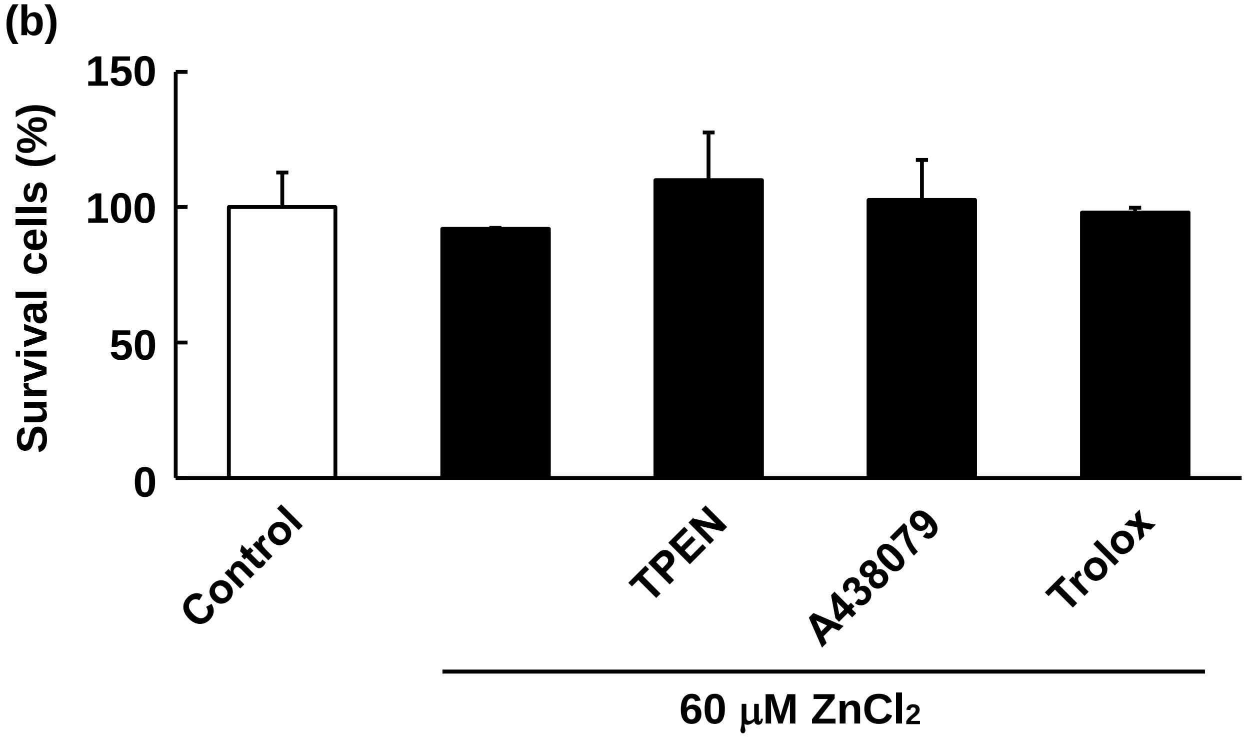

Supplementary Figure S6

Supplementary Figure S6

Microglial cell proliferation. After microglia had been treated with or without 1 μM TPEN for 30 min, 30 μM A438079 for 5 min, or 500 μM Trolox for 5 min, they were washed with warmed Eagle's minimum essential medium and incubated with 60-120 μM $ZnCl_2$ for 2 h. They were then treated with (**a**) or without (**b**) 1 ng/mL LPS for 22 h. The number of PI-negative cells was regarded as the number of living cells. Data are expressed as the mean ± the standard error of the mean (n = 3). *$p < 0.05$, significantly different from the control group. PI: propidium iodide; TPEN: *N*,*N*,*N'*,*N'*-tetrakis(2-pyridylmethyl)ethylenediamine; LPS: lipopolysaccharide.

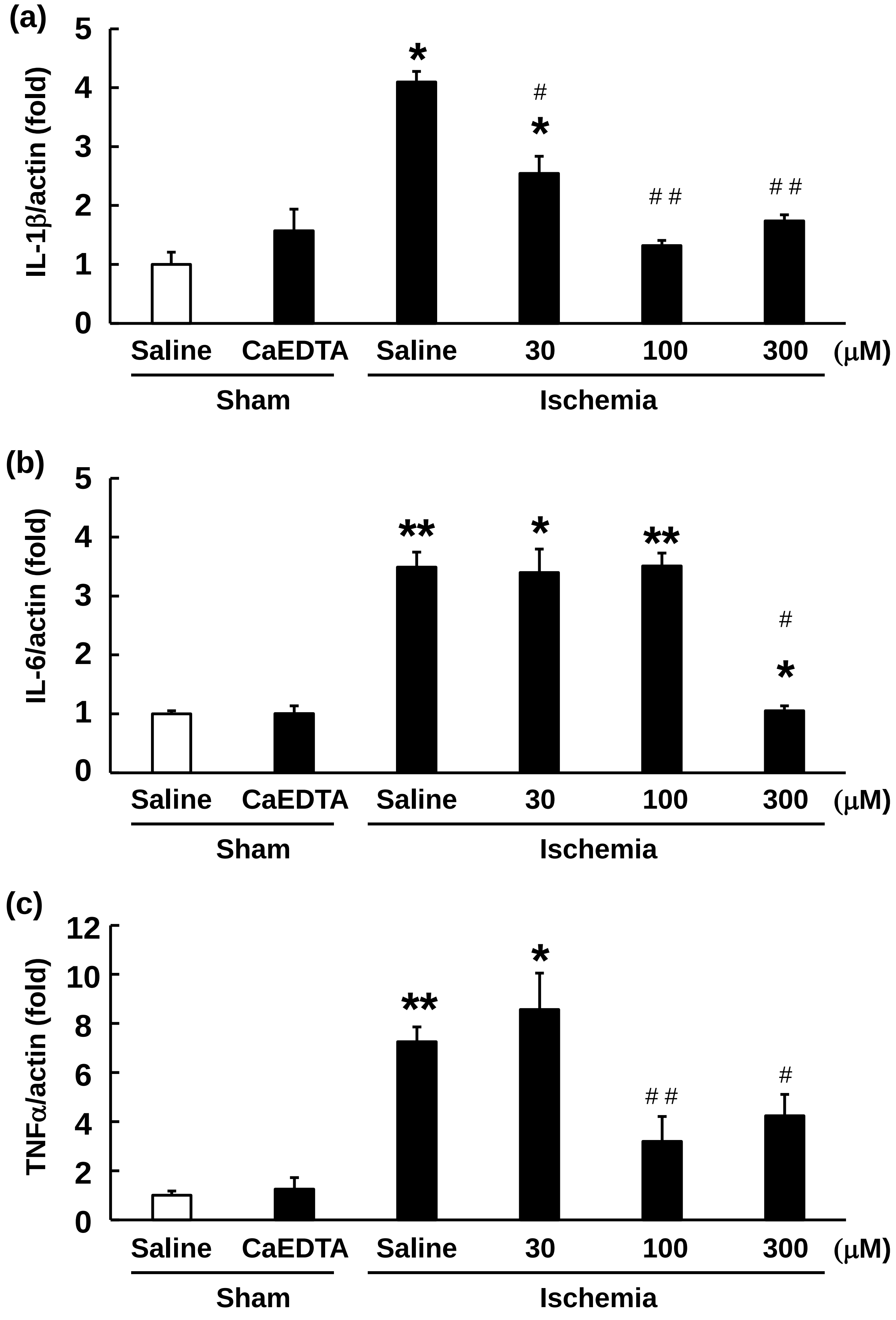

Supplementary Figure S7

Supplementary Figure S7

Dose-dependent attenuation of ischaemia-induced expression of IL-1β in the hippocampus following CaEDTA pre-administration. Mice were subjected to transient forebrain ischaemia 5 min after intraventricular injection of a zinc chelator, CaEDTA (30-300 mM in 2 μL volume). Real-time quantitative polymerase chain reaction was performed using total RNA extracted from the hippocampus of mouse brains 3 days after ischaemia. The amount of mRNA for interleukin-1 beta (IL-1β) (**a**), interleukin-6 (IL-6) (**b**), and tumour necrosis factor-alpha (TNFα) (**c**) was normalised to the amount of mRNA for β-actin. Data are expressed as the mean ± the standard error of the mean (n = 3). $^{*}p < 0.01$, significantly different from the vehicle-treated sham group; $^{\#}p < 0.05$, $^{\#\#}p < 0.001$, significantly different from the vehicle-treated ischaemic group.

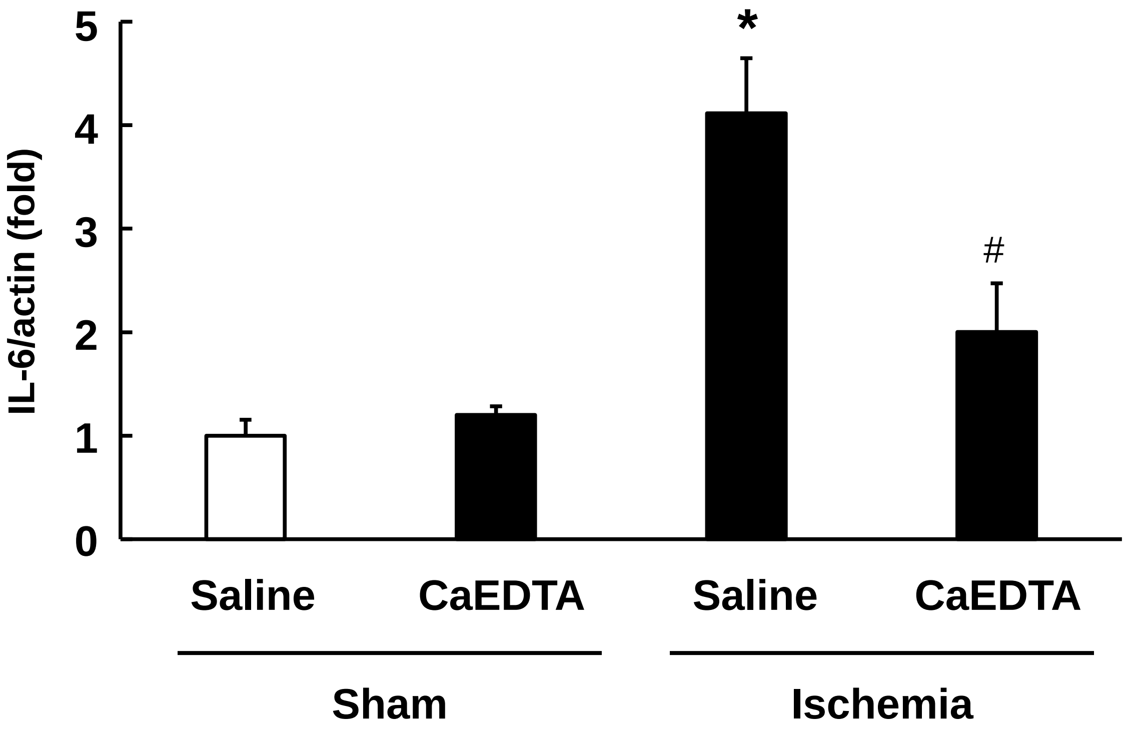

Supplementary Figure S8

Supplementary Figure S8

Effects of a zinc chelator on increased expression of IL-6 mRNA in the hippocampus 5 days after ischaemia. Mice were subjected to transient forebrain ischaemia 5 min after intraventricular injection of a zinc chelator, CaEDTA (300 mM in 2 μL volume). Real-time quantitative polymerase chain reaction was performed using total RNA extracted from the hippocampus of mouse brains 5 days after ischaemia. The amount of mRNA for IL-6 was normalised to the amount of mRNA for β-actin. Data are expressed as the mean ± the standard error of the mean (n = 4). $^{*}p < 0.05$, significantly different from the vehicle-treated sham group; $^{\#}p < 0.01$, significantly different from the vehicle-treated ischaemic group.

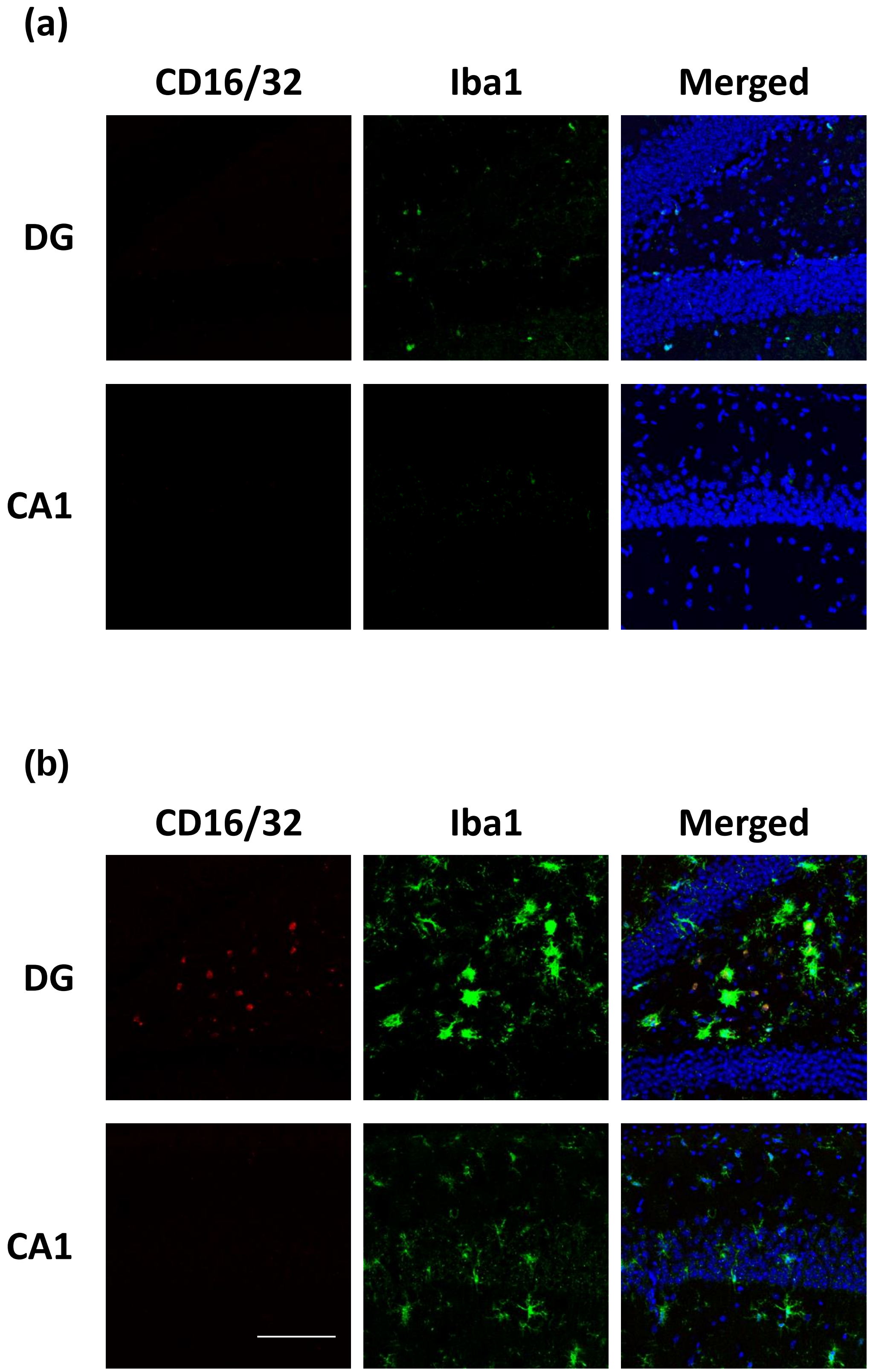

Supplementary Figure S9

Supplementary Figure S9

M1 activation of microglia in the dentate gyrus (DG), but not CA1, of the hippocampus after cerebral ischaemia. Mice were subjected to sham injury (**a**) or transient forebrain ischaemia (**b**). Representative images of fluorescent double staining of CD16/32 (red) and Iba1 (green) in the hippocampal DG and CA1 region 3 days after ischaemia. Nuclei were stained with DAPI (blue). Scale bar = 100 μm.

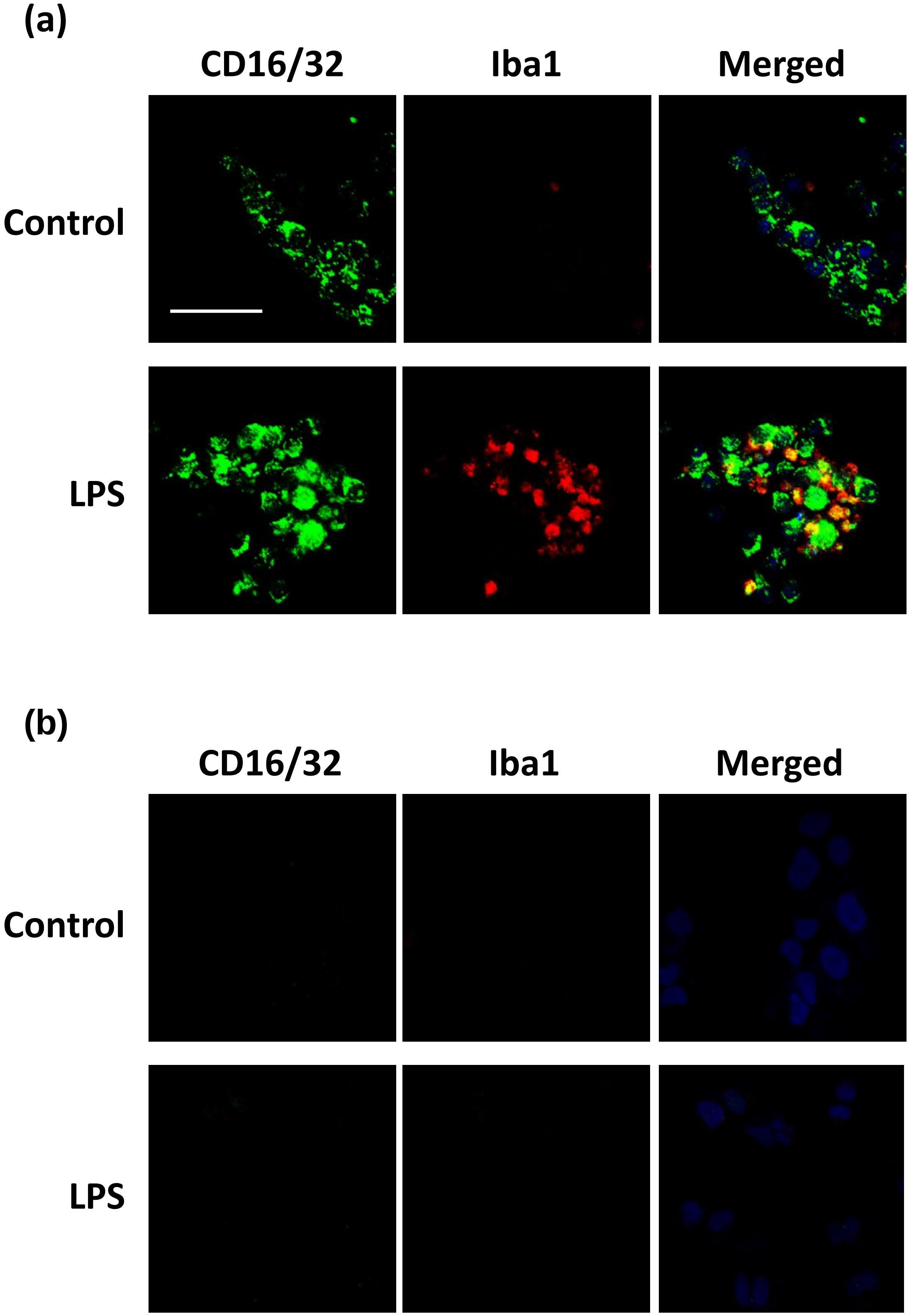

Supplementary Figure S10

Supplementary Figure S10

Verification of antibodies specificity. BV2 cells (**a**) and T98G cells (**b**) were treated with 100 ng/mL LPS for 48 h. Representative images of fluorescent double staining of CD16/32 (red) and Iba1 (green) in BV2 cells and T98G cells. Merged images depict CD16/32-positive signal (yellow). The nuclei were stained with DAPI (blue). Scale bar = 60 μm.

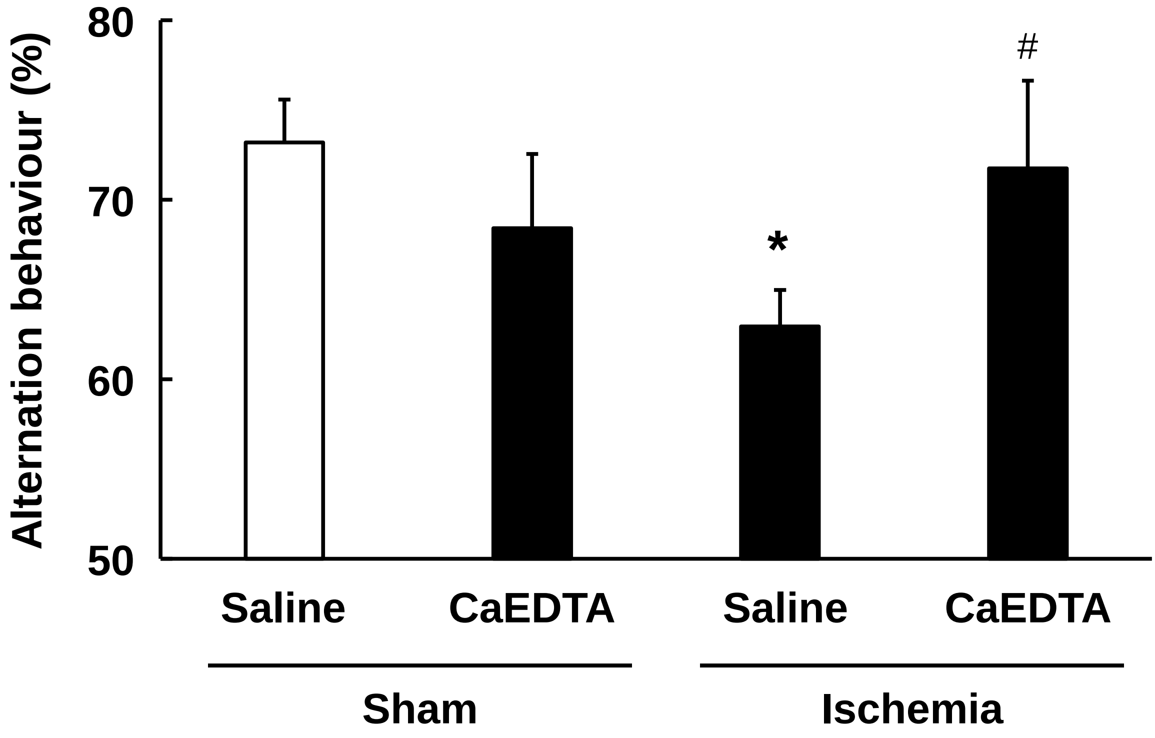

Supplementary Figure S11

Supplementary Figure S11

Prevention of ischaemia-induced short-term working memory deficits by CaEDTA pre-treatment. The Y-maze test was performed 5 days after transient forebrain ischaemia Percentage of alternation during a 10-min session in the Y-maze test was measured. Data are expressed as the mean ± the standard error of the mean (n = 5). *p < 0.01, significantly different from the vehicle-treated ischaemic group. #p < 0.05, significantly different from the saline-treated ischaemic group.
